# The Enterotoxicity of *Clostridium difficile* Toxins

**DOI:** 10.3390/toxins2071848

**Published:** 2010-07-14

**Authors:** Xingmin Sun, Tor Savidge, Hanping Feng

**Affiliations:** 1 Tufts Cummings School of Veterinary Medicine, North Grafton, MA, 01536, USA; Email: Xingmin.sun@tufts.edu; 2 The University of Texas Medical Branch, Galveston, TX, 77555, USA; Email: tcsavidge@utmb.edu

**Keywords:** *Clostridium difficile*, toxin A (TcdA), toxin B (TcdB), enterotoxicity

## Abstract

The major virulence factors of *Clostridium difficile* infection (CDI) are two large exotoxins A (TcdA) and B (TcdB). However, our understanding of the specific roles of these toxins in CDI is still evolving. It is now accepted that both toxins are enterotoxic and proinflammatory in the human intestine. Both purified TcdA and TcdB are capable of inducing the pathophysiology of CDI, although most studies have focused on TcdA. *C. difficile* toxins exert a wide array of biological activities by acting directly on intestinal epithelial cells. Alternatively, the toxins may target immune cells and neurons once the intestinal epithelial barrier is disrupted. The toxins may also act indirectly by stimulating cells to produce chemokines, proinflammatory cytokines, neuropeptides and other neuroimmune signals. This review considers the mechanisms of TcdA- and TcdB-induced enterotoxicity, and recent developments in this field.

## 1. Introduction

*Clostridium difficile* is a Gram-positive, spore-forming anaerobic bacillus. It is the most common cause of nosocomial antibiotic-associated diarrhea and is the etiologic agent of pseudomembranous colitis [1]. With the recent emergence of hypervirulent strains, the incidence of *C. difficile*-associated diarrhea and intestinal inflammatory disease (collectively designated CDI) has increased significantly in both North America and Europe, causing lengthy hospitalization, substantial morbidity and mortality. Of further concern is the recent emergence of hypervirulent strains that are resistant to antibiotics.

CDI is a toxin-mediated disease. Two exotoxins, toxin A (TcdA) and toxin B (TcdB), are the most extensively studied major virulence factors, supported by reports demonstrating that *C. difficile* clinical isolates lacking both toxin genes are non-pathogenic in humans and animals [2,3,4]. In addition to TcdA and TcdB, a limited number of *C. difficile* isolates also produce a binary toxin (CDT) that exhibits ADP-ribosyltransferase activity [5,6,7]. The pathophysiological role of CDT in CDI remains poorly understood [8,9,10]. In addition to these toxins, several other factors may play a role in disease manifestation. These factors include fimbriae and other molecules facilitating adhesion, capsule production and hydrolytic enzyme secretion, although none of these have been studied in detail [11,12,13]. Recent studies have also shown that the surface layer proteins of *C. difficile* play an important role in bacterial colonization, and that antibodies raised against these proteins are partially protective [14,15].

TcdA and TcdB possess a wide spectrum of biological activities, which may contribute to the range of symptoms associated with CDI. Toxin-induced disruption of the cytoskeleton leads to cytopathic effects in cultured cells within hours of intoxication, and this effect has been suggested to be mediated through Rac1 inactivation [16]. Although TcdB is generally more potent (~1000 fold) than TcdA, both are cytotoxic to most cultured cells where they trigger caspase-dependent apoptosis [17,18,19]. TcdA and TcdB also possess potent proinflammatory activity, and are capable of stimulating intestinal epithelial cells and immune cells to produce cytokines and chemokines [20,21,22,23,24]. Ileal-loop experiments in animal models have clearly demonstrated that TcdA is an enterotoxin [25,26]. TcdB was initially reported to exhibit no enterotoxic activity in animal models [27,28]. However, later studies have demonstrated its enterotoxic and proinflammatory activities in human colonic biopsies [29], human intestinal xenografts in immunodeficient (SCID) mice [20], and in hamsters [30]. To support this view, TcdA^−^B^+^ *C. difficile* clinical isolates can cause disease in patients and in experimental animal models [30,31]. Both TcdA and TcdB are highly toxic when administered systemically [32,33]. Systemic toxemia may therefore contribute to extraintestinal disease complications associated with severe cases of CDI [34,35]. In this review, we highlight the mechanisms of TcdA- and TcdB-induced enterotoxicity, and consider recent developments in this area.

## 2. *C. difficile* Infection in Humans

*C. difficile* infection is caused by the ingestion of vegetative organisms and spores, most likely the latter which survive exposure to gastric acidity and germinate in the colon [36,37]. Antibiotic exposure is the most significant risk factor in developing CDI [38]. The clinical manifestations are highly variable, ranging from asymptomatic carriage, to mild self-limited diarrhea, to severe pseudomembranous colitis (collectively designated as CDI). Systemic complications are rare, but have been reported [39,40,41,42,43]. Standard therapy depends on treatment with vancomycin and/or metronidazole, neither of which is fully effective [44]. More importantly, an estimated 15–35% of those infected with *C. difficile* relapse following treatment [45,46]. Alternative experimental treatment options include the use of probiotics, fecal treansplant, toxin-absorbing polymer, new antibiotics, monoclonal antibodies, IVIG, and toxoid vaccines [47,48,49,50,51,52]. 

*C. difficile* infection accounts for approximately 15–25% of cases of antibiotic-associated diarrhea, and the incidence of infection is rising steadily [53]. Several recent hospital outbreaks of CDI in North America, associated with high morbidity and mortality rates, have been attributed to the widespread use of broad-spectrum antibiotics. The emergence of new and more highly virulent strains of *C. difficile* has also contributed to the increased incidence and severity of the disease [54,55]. 

Asymptomatic carriage of *C. difficile* in infants is estimated to be approximately 50% or higher [56]. These infants can have high numbers of toxigenic *C. difficile* and high levels of toxins in their stools without showing symptoms [57,58,59,60,61]. It is unclear why such infants are usually refractory to CDI, even though they carry high numbers of the organism and toxins. Fetal intestinal epithelial cells were reported to be much less sensitive to intoxication than adult cells, and this may contribute to this asymptomatic carriage [62]. Another hypothesis is that infants may lack the membrane receptors required for toxin binding. Alternatively, these receptors may be masked by a thicker layer of mucus in infants, supported by the observation that mucins directly inactivate *C. difficile* toxins [63].

## 3. Animal Models of CDI

*In vivo* animal infection modes for CDI have been established using a number of animal species, including hamsters, guinea pigs, rabbits, germfree mice and rats, but most of the work has been done in hamsters [64,65,66,67,68,69,70,71,72,73]. The disease in hamsters can be induced by a variety of antibiotics, and the intestinal damage is mainly localized to the cecum, with some involvement of the ileum. Infected hamsters develop fulminant diarrhea and die rapidly from severe enterocolitis. Lung damage has also been reported in hamsters [28]. Because of this fulminant disease course, the hamster model does not accurately recapitulate the disease course and progression in humans. Chen and colleagues recently developed a conventional mouse model of CDI, in which the primary injury site is the colon. Moreover, the disease progression follows a similar pattern to human CDI [74]. Our group has also recently established a piglet model of acute and chronic *C. difficile* infection, which mimics many of the key characteristics observed in human CDI [35]. Interestingly, in other less well defined animal models, TcdB was reported to cause damage and edema in cardiac tissue of zebrafish embryos [28,32], and severe jejunal lesions in rabbits [75].

In animal models, an unusual observation with *C. difficile* infections is that infant hamsters are insensitive to *C. difficile* infection [76,77], a phenomenon that is also seen in human infants. By contrast, neonatal gnotobiotic piglets are highly sensitive to *C. difficile* infection [35]. 

Studies in animal models have contributed greatly to our understanding of the pathogenicity of TcdA and TcdB. It was speculated that TcdA was the most important component in disease production because it elicits extensive tissue damage and fluid accumulation in several animal models [28,78,79,80]. Moreover, some reports showed that immunization with TcdA induces full protection in rabbits [81], hamsters [82] and gnotobiotic mice [83] against *C. difficile* infection. Serum TcdA and TcdB titers also correlate with disease activity in CDI patients [84,85,86]. In contrast, TcdB does not exhibit enterotoxicity in rabbit ileal and colonic loops [78], hamster ileal loops [87] or in mouse ileal loops [88] even though it is a more potent cytotoxin than TcdA [89]. However, *ex vivo* studies using human colonic explants showed that TcdB is more potent than TcdA in inducing epithelial barrier damage and proinflammatory cytokines signaling, consistent with enterotoxicity [29]. We have confirmed this finding *in vivo* in a human intestinal xenograft model [20]. In support of this, TcdA^−^TcdB^+^ strains are known to cause human CDI, with a full spectrum of disease manifestations [90]. TcdA^−^TcdB^+^ strains can also cause disease in rabbit, hamster and mouse models [91,92,93]. More recently, Lyras *et al.* generated isogenic *C. difficile* (strain 630) in which the *tcdA* or *tcdB* gene is insertionally inactivated. In this study, the isogenic strains with only intact *tcdB* gene, but not *tcdA*, caused CDI in hamsters [30]. Taken together, it is believed that both TcdA and TcdB are enterotoxic and responsible for the full spectrum of symptoms in CDI. Consistent with this view, several studies have shown that antibodies against both TcdA and TcdB are necessary to protect hamsters from CDI [94,95]. 

Why TcdB does not show enterotoxicity in ileal loops of animal models, while TcdA^−^TcdB^+^ *C. difficile* strains are able to cause disease in the same animals is still unknown. It was reported that hamster and rabbit intestine possess specific TcdA brush border receptors [96,97], while receptors for TcdB have not yet been identified. One assumption is that TcdB receptors do not exist in rodent small intestine, but might be located in the colon or cecum. Alternatively, TcdB could be chaperoned by another as yet unknown bacteria-derived protein, although, purified TcdB has been shown to cause histological damage in hamster caeca [87,98]. Moreover, the disease in hamsters is localized in the cecum with some involvement of ileum. Ironically, even though the disease in mice and rats involves in colon [74,77], most of the gut-loop experiments have been carried out using ileum segments. Therefore, intestinal activities of TcdB in animal models should be carefully interpreted and studies testing TcdB enterotoxicity in cacecal loops of rabbits or colonic loops of mice are urgently needed. Secondly, using transwell system TcdB has been indicated to preferably bind to basal surface of epithelial monolayers [99]. Therefore, TcdB receptors may not be exposed apically to the gut lumen. Once the epithelial barrier is impaired by other factors, TcdB may gain access to its “receptors”, leading to intestinal inflammation. In support of this view, it was reported that when given intragastrically to hamsters, TcdB did not cause any disease unless the intestine is first breached either by TcdA or by manipulation [80]. 

## 4. Mechanism of Action and Functional Domains of TcdA and TcdB

### 4.1. Structure of TcdA and TcdB

**Figure 1:**
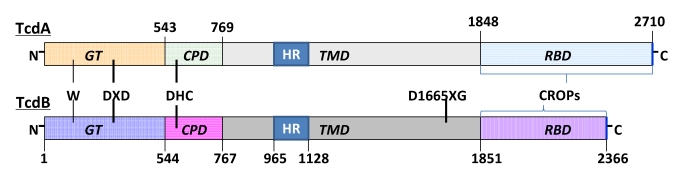
Structure of TcdA and TcdB. TcdA and TcdB consist of four domains: an *N-*terminal glucosylatransferase domain (GT), an autocatalytic cysteine protease domain (CPD), a central translocation domain (TMD) covering a hydrophobic region (HR), and a *C-*terminal receptor binding domain (RBD) consisting of clostridial repetitive oligopeptides (CROPs). The DXD (Asp-X-Asp) motif and a conserved trytophan present in the GT domain are involved in Mn^2+^ and UDP-glucose binding. The conserved DHC (Asp-His-Cys) catalytic triad in the CPD domain mediates toxin autocleavage. The DXG (Asp-X-Gly) motif in the TMD region of TcdB was reported to possess aspartate protease activity, which could be involved in toxin cleavage.

TcdA (308 kDa) and TcdB (269 kDa) belong to the Clostridial glucosylating toxins family, and share high amino acid sequence identity [100]. They are structurally similar to each other [89], including an *N*-terminal glucosyltransferase domain (GT), the newly identified autocatalytic cysteine proteinase domain (CPD), the central translocation domain (TMD) covering a hydrophobic region (HR), and the *C-*terminal receptor binding domain (RBD) consisting of clostridial repetitive oligopeptides (CROPs) [101] (Figure 1). Recently, 3D structures of two RBD fragments of TcdA, CPD of TcdA, and the catalytic domain (residues 1–543) of TcdB have been solved [102,103,104,105]. However, due to their unusual sizes and linear domain arrangement the high-resolution structures of holotoxins have not been determined. Most recently a group of scientists used small-angle X-ray scattering (SAXS) methods to obtain low-resolution structure/model of native TcdB [106]. The structure showed a monomeric shape of TcdB in solution. They modeled the structures of individual domains onto the SAXS structure of TcdB and obtained its 3D structure, which shows four distinct structure domains separating from each other. Structures of GT, CPD and TMD can be aligned within three domains of the SAXS structure, but the TMD appears as a large solvent-exposed protrusion [106].

### 4.2. The C-terminal receptor binding domain (RBD) mediates receptor binding

The most striking feature of the RBDs of TcdA and TcdB is the presence of CROPs of 21-, 30-, or 50-amino acid residues. Different approaches to analyzing the sequence of CROPS of TcdA reveal that this region contains between 30 and 38 contiguous repeats, whereas in TcdB those appear to be between 19 and 24 residues [102,103]. CROPs may play a putative role in initial target cell interaction and receptor binding. Recently, two RBD fragments (terminal 127 and 255 residues) of TcdA were crystallized [102,103]. These studies showed that TcdA folds in a solenoid-like structure, which is suggested to increase the surface area of proteins and enable protein-protein or protein-carbohydrate interactions. It was previously showed that TcdA binds to the trisaccharide Galα1-3Galβ1-4GlcNAc [107], carbohydrate antigens like Lex and Ley [108], components in human milk and carbohydrates from brush border membranes of hamster ileum [109]. Interaction of TcdA with glycosphingolipids has been suggested as well [110]. Recently, the crystal structure of TcdA was solved in complex with a synthetic carbohydrate consisting of Galα1-3Galβ1-4GlcNAc structure [103]. However, human do not have a functional α-galactosyltransferase and, therefore, cannot form α-galactosyl bonds, indicating that the carbohydrate structure Galα1-3Galβ1-4GlcNAc cannot be part of intestinal receptors in human [111,112]. Therefore the disaccharide Galβ1-4GlcNAc, which is present in humans, has been suggested to be part of a possible receptor. Until recently, it was reported that glycoprotein 96 (gp96) is a human colonocyte plasma membrane binding protein for TcdA [113]. Our understanding on the TcdB receptor is even more limited. Published data indicate that TcdA and TcdB use different types of receptors. The receptor for TcdB appears to be basolateral, whereas the TcdA receptor is on the apical sites [114].

### 4.3. The central translocation domain (TMD), autocatalytic cysteine proteinase domain (CPD) and uptake of TcdA and TcdB

The putative TMD makes up more than 50% of the total size of the toxins and contains a hydrophobic region which is most probably responsible for membrane insertion. To date, little is known about exact functions of the TMD. However, recently it was suggested that the Asp-Xaa-Gly (DXG) motif of TcdB possesses aspartate protease activity, which could be involved in toxin cleavage [115].

The newly identified CPD domain is located between the GT and TMD domains, starting from residue 543 to residue 767 in TcdB. The CPD domain contains a conserved catalytic triad (Asp587-His653-Cys698) of a cysteine protease and mediates toxin autocleavage. Interaction between the RBD and the host cell receptors is believed to initiate receptor-mediated endocytosis [108,116,117]. Although the intracellular mode of action remains unclear, it has been proposed that the toxins undergo a conformational change triggered by the low pH environment ofthe endosomal compartment, leading to a membrane insertion and channel formation [118,119,120,121]. A host cofactor, inositol *hexakis*phosphate (InsP6), is thought to be the cofactor to trigger CPD-mediated autocatalytic cleavage of the toxins, and the subsequent release of the *N-*terminus GT domain into the cytosol [115].

### 4.4. N-terminal glucosylatransferase domain (GT)

The *N* terminus (residues 1–543) harbors the glucosyltransferase activity of the toxins and is the biologically activity domain. The crystal structure of the GT domain of TcdB revealed a set of essential amino acid residues involved in glucosyltransferase reaction or substrate binding [104]. The Asp-Xaa-Asp (DXD) motif and a conserved tryptophan are involved in Mn^2+^ and UDP-glucose binding. In addition to the DXD motif, several other residues are also essential for enzyme activity [122].

### 4.5. Small GTPase proteins as targets of TcdA and TcdB

The common features of small GTPases include their small molecular mass (18–28 kDa), their *C-*terminal polyisoprenylation, and their property of binding to guanine nucleotides. They are molecular relays, which transmit signals when bound to GTP and stop doing so when bound to GDP. Small GTPases are subdivided into the subfamilies of Ras, Rho, Rab, Arf, and Ran. Rho subfamily members also called Rho GTPases or Rho proteins are major known intracellular targets of TcdA and TcdB. Approximately 20 Rho GTPases have been described, including RhoA, Rac1 and Cdc42 [100]. 

Once in the cytosol, the GT effector domain of the toxins mono-O glucosylates low molecular weight GTPases of the Rho family, including RhoA, Rac1, and CDC42 [123]. Rho GTPases are inactive in the GDP-bound form and associated with guanine nucleotide dissociation inhibitors (GDI), which keep the GTPases in the cytosol. Guanine nucleotide exchange factors (GEFs) activate Rho GTPases, enabling interaction with different effectors to control numerous signaling processes. The active state of Rho GTPase is terminated by hydrolysis of bound GTP facilitated by GTPase-activating proteins (GAP). Rho GTPases regulate many host cell functions including epithelial barrier functions, immune cell migration, adhesion, phagocytosis, cytosecretion and immune cell signaling [100,101]. Glucosylation of Rho proteins inhibits their “molecular switch” function, thus blocking Rho GTPase-dependent signaling. This leads to alterations in the actin cytoskeleton, loss of tight junction integrity, massive fluid secretion, acute inflammation and necrosis of the colonic mucosa [26,123,124]. In addition to TcdB and TcdA from the prototype strain VPI-10463, toxin variants of *C. difficile* toxin B, TcdB-1470 and TcdB-8864, have been isolated from TcdA-negative strains [125,126]. These variants have been reported to produce a somehow different cytopathic effect (CPE) than the classical neurite-like morphology induced in fibroblasts by TcdB-10463. TcdB-1470 was shown to be a functional hybrid possessing the receptor-binding and internalization domain of TcdB-10463 and the glucosyltransferase domain of *C. sordellii* TcsL. TcdB-1470 does not glucosylate RhoA but predominantly modifies Rac1 from Rho subfamily and also members of the Ras subfamily (Ras, Rap, Ral). TcdB-8864 has also altered glucosylation specificity and recognizes Rac1, Rap2 and Ra1. Another variant TcdB, TcdB-C34 has substrate specificity on Rho, Rac and Cdc42 from Rho subfamily and Ras, Ral, and Rap from Ras subfamily [100,127].

*C. difficile* is a very heterogenerous species. Currently, 24 variant toxinotypes are identified in addition to reference strain VPI 10463. The most-studied variant strains are TcdA^−^ TcdB^+^ strains and binary toxin CDT-producing strains, which will be discussed below. Variant toxins show differences in size, antibody recognition, glucosylation of small GTPases and consequently in their effects on the cells [128].

### 4.6. C. difficile binary toxin (CDT)

In addition to TcdA and TcdB, some *C. difficile* isolates (less than 10%), including the epidemic NAP1/027 strain (toxinotype III), produce a third toxin called *C. difficile* binary toxin (CDT) [129]. Most binary toxin-positive strains also producedTcdB and/or TcdA. However, they had significant changes in the *tcdA* and *tcdB* genes and belonged to variant toxinotypes III,IV, V, VII, IX, and XIII [129]. Strains that produce CDT but neither of the TcdA and TcdB have been recently described [9,130]. This toxin is a two-component ADP ribosyltransferase encoded by the genes *cdtA* (enzymatic component) and *cdtB* (binding component). CDT-positive isolates are cytopathic in cell culture. When tested in CDI animal models, TcdA^−^TcdB^−^CDT^+^ strains cause fluid accumulation in rabbit ileal loops but no diarrhea or death in hamster models [9]. A more recent study showed CDT induces formation of microtubule-based protrusion and increases adherence of bacteria to intestinal epithelium [10]. Another exciting advancement is the determination of CDTa in complex with ADP ribose donors, NAD and NADPH [131]. This structure will have implications in understanding CDT recognition, and will be valuable for the rational design of therapeutic strategies.

## 5. The Mechanisms Underlying the Enterotoxicity of TcdA and TcdB

The clinical appearance of CDI is highly variable, ranging from mild self-limited diarrhea to severe pseudomembraneous colitis. The disease starts in the large bowel and shows distinguishing “volcanic eruption” characteristics of the pseudomembranous lesion observed in severe colitis. Diarrhea and colitis are two major clinic manifestations of CDI. In severe cases of CDI, patients present with systemic inflammatory syndromes that include abdominal pain, fever, hypotension, tachypnea, and leukocytosis. Most of these clinical presentations can be ascribed to direct intestine tissue damage and inflammation mediated by TcdA and TcdB. This section describes how TcdA and TcdB can induce such intestinal damage and inflammation. The proinflammatory mediators involved in the enterotoxicity of TcdA and TcdB are summarized in Table 1.

**Table 1:** Proinflammatory mediators involved in the enterotoxicity of TcdA and TcdB.

Proinflammatory mediators	Sources (cells or tissue)	Known or proposed functions	References
IL-8	Intestinal epithelia, macrophages, peripheral blood monocytes	Neutrophil recruitment	[21,23,132]
GRO-α (growth-related oncogene)	Intestinal epithelia	Neutrophil recruitment	[21,133]
MIP-1 (macrophage inflammatory protein-1)	Intestinal epithelia, macrophages	Neutrophil recruitment	[133,134]
MIP-2 (macrophage inflammatory protein-2)	Intestinal epithelia, macrophages	Neutrophil recruitment	[133,134]
ENA-78 (epithelial neutrophil-activating peptide-78)	Intestinal epithelia	Neutrophil recruitment	[21,133]
MCP-1 (monocytes-chemotactic protein-1)	Intestinal epithelia	Neutrophil recruitment	[21]
ICAM-1 (intercellular adhesion molecule-1)	Endothelial cells, neutrophils, epithelia	Neutrophil adhesion to endothelial cells	[135,136,137]
IL-1	Macrophages, dendritic cells,	Neutrophil recruitment, enhancing IL-8 production,	[135,136,138,139]
IL-6	Monocytes, dendritic cells	not specified	[24]
TNF-α	Macrophages, monocytes	Neutrophil recruitment	[24,138,139]
IFN-γ	Neutrophils	Enhancing chemokine and ICAM-1 expression expression	[134]
LB4 (Leukotriene B4)	Macrophages, mast cells	Neutrophil recruitment, activation of transient receptor potential vailloid (TRPV1) and SP release	[138,140,141]
LC4 (Leukotriene C4)	Mast cells	Stimulation of fluid secretion by intestinal epithelia	[140]
RMCP II (rat mast cell protease II)	Mast cells	Indicator of mast cell activation	[140]
SP (substance P)	Intestinal neurons	Activation of mast cells and macrophages	[142,143]
CGRP (calcitonin gene-related peptide)	Intestinal neurons	Activation of mast cells and macrophages	[142,143]
NT (neurotensin)	Intestinal neurons	Activation of mast cells	[144]
VIP (vasoactive intestinal polypeptide)	Colonic submucosal neurons	Activated partially via an IL-1β-dependent pathway ( its role in the intestine is to increase motility	[145]
PAR2 (protease activated receptor 2)	Enterocytes, neurons, endothelial cells, neutrophils	Intestinal inflammation	[146]
Inflammasome	Macrophages	IL-1β production	[147]
Melanin-concentrating hormone (MCH)	Intestinal tissue	Upregulation of IL-8 transcription	[148]
Reactive oxygen species (ROS)	Neutrophils	Direct damage of proteins and lipids, induction of IL-8 and ICAM-1	[149,150,151,152]
Cyclooxygenase-2 (COX-2)	Human colonocyte, human intestinal xenograft	Formation of PGE2	[150,153]
Prostaglandin E2 (PGE2)	Human colonocyte, human intestinal xenograft	Intestinal inflammation	[150]
Phospholipase A2	Rabbit ileal tissue, human T-84 cells	Synthesis of inflammatory lipid mediators	[154]
Platelet-activating factor (PAF)	Rabbit ileal tissue	Stimulation of fluid secretion by intestinal epithelial cells	[155]
Na+/H+ exchanger (NHE)	Intestinal epithelial cells	Involvement in Na+ absorption and fluid homeostasis	[156]
Angiotensin II (ANGII)	Rabbit ileal tissues	Regulation of intestinal secretion and absorption	[157]
Epidermal growth factor receptor (EGFR)	Human colonic epithelial cells	Activation of IL-8	[158]

### 5.1. Disruption of the tight junctions of epithelial barriers

The direct target tissue of the *C. difficile* toxins is the colonic epithelium. One of the most direct events attributed to TcdA and TcdB in the intestine is their ability to disrupt tight junctions and to breach the intestinal epithelial barrier [159]. Inactivation of Rho proteins by the toxins causes the loss of epithelial barrier function, since these small GTPases are critical regulators of tight junction function. TcdA and TcdB both catalyze the transfer of glucose from UDP-glucose to threonine-37 in RhoA and threonine-35 in Rac1 and Cdc42, inactivating these signaling proteins [160]. In human colonic epithelial cell lines and in human colonic explants, both toxins impair tight junction function resulting in a loss of electrical resistance and the peri-junctional actinomyosin ring, and an increased paracellular permeability from the luminal domain [29,161,162,163,164]. Increased blood-to-lumen permeability is also observed *in vivo* in rat and rabbit intestine exposed to TcdA [165,166]. This increase in paracellular permeability was reported to be Rho-independent, albeit via an unknown mechanism [167]. However, Teicher *et al.* found that treatment of Caco-2 cells with glucosyltransferase-deficient TcdA does not significantly induce decrease in transepithelial electricalresistance (TER), strongly arguing for a critical role of Rho glucosylation in this process [168]. The enhanced mucosal permeability causes an array of consequences, including promoting access of neutrophils to the intestinal lumen as is seen in CDI patients. In addition to disruption of tight junctions, TcdA also causes detachment of epithelial cells in human colonic mucosal explants [29]. Adhesion of epithelial cells to the underlying matrix occurs by focal contact formation. More recently, Kim and colleagues described mechanisms on how TcdA induce detachment of epithelial cells [169]. They found that TcdA binds colonocyte Src, causing dephosphorylation of the focal adhesion kinase (FAK) and paxillin, which is independent of the Rho glucosylation [169].

### 5.2. Apoptosis and necrosis of epithelial cells and other cell types

The apoptogenic properties of TcdA and TcdB are well known, and they may contribute to the inflammatory process induced by these toxins. Treatment of intestinal epithelial cell lines with either TcdA or TcdB results in their apoptosis [18,19,132,170]. TcdA and TcdB induce both apoptosis and necrosis of epithelial cells in human intestinal xenografts in a chimeric mouse model [20]. TcdA also induced apoptosis in tissue cultures of human colonic biopsy specimens [132]. Moreover, TcdA and TcdB may gain access to the underlying lamina propria and submucosa, to act directly on other cell types once the epithelial barrier is breached. In fact, TcdA-induced apoptosis of monocytes and T cells is also reported [171,172].

Brito and his colleagues reported that TcdA-induced apoptosis of T84 cells is completely inhibited by blocking toxin enzymatic activity on Rho GTPases with uridine 5'-diphosphate-2',3'-dialdehyde, and is partially inhibited by caspase-1, -3, -6, -8, and -9 inhibitors [18]. Caspases 3, 6, 8, and 9, and Bid are activated following cellular intoxication. TcdA also induces changes in mitochondrial membrane potential and release of cytochrome c after 18-24 h of intoxication, a time course that is similar to caspase-9 activation. The authors concluded that TcdA induces apoptosis by a mechanism that depends on Rho-inactivation and the subsequent activation of caspases 3, 6, 8, and 9, as well as Bid and mitochondrial damage followed by cytochrome c release. Using glucosyltransferase-deficient mutants, Gerhard and his colleagues demonstrated that the activation of caspases and induction of apoptosis in human colonic HT29 cells depend on the glucosyltransferase activity of TcdA [17]. In a related study, exposure of human umbilical cord vein endothelial cells (HUVEC) to TcdB 10463 (which inhibits RhoA/Rac1/Cdc42), or to C3 toxin (which inhibits RhoA, B, and C), resulted in apoptosis, whereas inactivation of only Rac1/Cdc42 with TcdB-1470 was unable to induce apoptosis, suggesting that RhoA inhibition is responsible for apoptosis in endothelial cells [173,174].

Glucosyltransferase-independent apoptosis was reported as well. Matarrese and colleagues demonstrated that TcdB causes apoptosis in human epithelial HEp-2 cells by directly acting on mitochondria, which does not require the *N-*terminal Rho-inhibiting activity of the toxin [175]. However, the authors treated purified mitochondria with TcdB or the GT fragment, which is not the case in cells. Moreover, the purity of the toxins was not demonstrated. It was found that treatment of cells with TcdA leads to an accumulation of the toxins at the mitochondria within five min following exposure, and this event occurs before detectable glucosylation activity of Rho proteins [151]. However, a contrary finding was reported that mitochondrial damage as the initiation of apoptosis started after 18–24 h of toxin treatment and was clearly Rho-dependent [18]. Another study compared apoptosis induction in HeLa cells by a TcdB fragment containing only GT domain versus the intact holotoxin B [19]. Holotoxin B induces apoptosis via activation of caspase 3, whereas the GT domain does so in a caspase-independent pathway [19]. The authors concluded that TcdB triggers caspase-dependent apoptosis as a result of substrate inactivation and may evoke caspase-independent apoptosis due to a second, yet undefined activity of TcdB. However, in this work, GT domain was either delivered as anthrax lethal toxin chimera or expressed in the cells, which may not mimic the finding that only the catalytic domain is cleaved and delivered to the cytosol [176].

In addition to apoptosis, necrosis is also likely to contribute to mucosal damage. For instance, TcdA has been shown to induce cell necrosis [171,177,178], and toxin-induced proinflammatory mediators may trigger epithelial necrotic death as discussed below. 

The roles of transcription factors in *C. difficile* toxins-induced apoptosis or necrosis were rarely described [177,179]. One report described that p38 was involved in both apoptosis and necrosis. However, transcription factors may also play protective roles in the host in response to *C. difficile* toxins. Chae *et al.* reported that IқB-kinase β (IKKβ) deficient mice exhibit a rapid and significant increase in intestinal epithelial apoptosis, acute mucosal inflammation, mucosal injury, luminal fluid secretion, and bacterial translocation in response to TcdA exposure in mouse ligated ileal loops [180]. Therefore, in addition to its roles in regulating secretion of chemokines or other proinflammatory events, NF-қB plays an important host mucosal protective role in response to TcdA. Recently, Hirota *et al.* have implicated an innate protective role for hypoxia-inducible factor (HIF-1) in response to TcdA [181]. In this report, deletion of intestinal epithelial HIF-1alpha was demonstrated to exhibit more severe, toxin-induced intestinal injury and inflammation in mice. In contrast, stabilization of HIF with chemicals attenuated toxin-induced injury and inflammation. 

### 5.3. The role of chemokines released by epithelial cells

An intense inflammatory response with a marked neutrophil accumulation is a key characteristic of the clinical pathophysiology of CDI [182,183]. In animal models, TcdA causes fluid secretion, mucosal edema and villus damage by inducing massive acute inflammation with neutrophil infiltration [183,184]. The mechanism by which neutrophils are recruited to sites of inflammation is a complex and multistep phenomenon that involves the expression of leukocyte and endothelial cell adhesion molecules, followed by neutrophil attachment and adhesion to the endothelium, and finally transmigration of neutrophils into the intestine mucosa [185]. These events are driven by a local production of a wide range of chemoattractants, and by activating cytokines that establish a chemotactic gradient and induce the expression of adhesion molecules in both endothelial cells and neutrophils. Several molecules are involved in neutrophil recruitment, including IL-8, epithelial neutrophil-activating peptide-78 (ENA-78), and the growth-related oncogene (GRO) family in humans [186]. Several reports have showed that intestinal epithelial cells can produce chemokine IL-8, GRO-α, ENA-78, and monocytes-chemotactic protein (MCP)-1 in response to TcdA [21,132,151,183]. IL-8 is a potent proinflammatory chemotactic factor that predominantly exerts its effects on neutrophils [187,188]. Interestingly, following intoxication of polarized Caco-2 colonocytes in a transwell system with TcdA, IL-8 and other chemokines are released predominantly into the basolateral compartment [21]. The same phenomenon was also observed by Canny and colleagues [137]. Macrophage inflammatory protein (MIP)-2 is the rat homologue of human GRO. TcdA induces MIP-2 release from intestinal epithelial cells which contributes to neutrophil transmigration in TcdA-induced enteritis [133]. At present, it is not entirely clear how the *C. difficile* toxins induce chemokine or cytokine (see below) production, as is reflected in several conflicting publications. Both Rho-dependent and Rho-independent cytokine release mechanisms have been proposed [177,189,190]. Critical involvement of nuclear factor-κB (NF-κB) or mitogen-activated protein kinase p38 in the cytokine secretion has also been described [133,177,191,192]. It will be interesting to see whether similarities exist in the signaling pathways initiated by TcdA versus TcdB. Certainly, differences have been reported for the two toxins, for example NF-κB is not involved in TcdB-mediated IL-8 production, whereas NF-κB plays an important role in TcdA-mediated IL-8 secretion [151,191].

### 5.4. Immune cells and proinflammatory cytokines/mediators

In addition to chemokines, release of proinflammatory cytokines or other mediators from immune cells seems to be a critical activation event in TcdA-mediated inflammation [25,193]. These proinflammatory cytokines are known to exert potent proinflammatory and cytotoxic effects. They also mediate septic shock and induce the acute-phase reaction. These mediators may promote proinflammatory and cytotoxic effects in the pathophysiological mechanisms of CDI, or they may act synergistically with the direct cytopathic effects of TcdA or TcdB. In fact, local release of TNF-α in the intestinal mucosa is known to cause cellular alterations, increase intestinal permeability, activate endothelial cells, and enhance inflammation [194]. IL-1β was reported to increase both IL-8 secretion by HT-29 cells exposed to TcdA, and promote intercellular adhesion molecule-1 (ICAM-1)-dependent neutrophil adhesion [135,136].

Neutrophil-derived proinflammatory mediators act on epithelial cells, causing destruction and necrosis of enterocytes and colonocytes [166]. Neutrophils contribute significantly to tissue damage, as these cells contain a potent arsenal of oxidants and proteases in azurophillic granules [25,133]. In addition to chemoattractant-induced recruitment of neutrophils in CDI, TcdA also directly interacts with neutrophils via a G protein-linked receptor. TcdA directly stimulates human neutrophils, promoting a transient increase in intracellular Ca^2+^ levels that stimulates neutrophil chemotaxis [25,195]. A blocking antibody to CD18, an ICAM-1 ligand in neutrophils, markedly reduces neutrophil infiltration in TcdA-exposed rabbit ileal loops [25]. Inhibition of neutrophil recruitment in this model is associated with a substantially reduced intestinal permeability, fluid secretion, and mucosal injury. Ishida and colleagues reported the essential involvement of IFN-γ in TcdA-induced enteritis [134]. Injection of TcdA into mouse ileal loops induces massive fluid secretion, disruption of intestinal mucosal integrity with neutrophil infiltration, and production of IFN-γ, TNF-α, MIP-1 and -2, keratinocyte-derived chemokine (KC), and ICAM-1. IFN-γ protein is mainly detected in infiltrating neutrophils. In contrast, TcdA-treated IFN-γ knockout mice show marginal histopathological changes and cytokine/chemokine gene expression is drastically attenuated. Furthermore, Ishida *et al.* demonstrated that pretreatment of wild type mice with a neutralizing anti-IFN-γ antibody prevents TcdA-induced enteritis. Therefore, locally produced IFN-γ may enhance neutrophil transmigration directly and/or indirectly by enhancing chemokine and ICAM-1 expression.

Intestinal mast cells are critically involved in neutrophil activation in TcdA-mediated inflammation. Increased mucosal and circulating levels of the specific mucosal mast cell enzyme, rat mast cell protease II (RMCP II), and degranulation of mucosal mast cells are observed within 15–30 min after TcdA exposure [107]. Depletion of mast cells significantly diminishes intestinal inflammation after TcdA exposure, and is restored following reconstitution of mast cells in mast cell-deficient mice [193]. In another study, stabilizing mucosal mast cells with ketotifen reduced the severity of secretory diarrhea and inflammation. Ketotifen inhibits TcdA-induced release of mast cell mediators, including leukotriene B4 and C4 and RMCP II [140]. Direct effects of TcdA and TcdB on mast cells was shown in rat basophilic leukemia (RBL) cells, murine peritoneal mast cells, and more recently in human mast cells HMC-1 [196,197,198]. Moreover, TcdB stimulates HMC-1 cell degranulation and p38 MAPK-dependent up regulation of IL-8 secretion [198]. 

Dendritic cells (DCs) are antigen-presenting cells that play important roles in innate and adaptive immune responses [199]. In addition to their role in antigen sampling and processing, DCs are potent immune regulatory cells that secrete a vast spectrum of modulating substances including chemokines and cytokines [200]. Recently, significant progress has been made in demonstrating that a subset of intestinal lamina propria DCs (CD11c^+^ and CD11b^+^) directly protrudes dendrites crossing epithelial barriers into intestinal lumen to sample bacterial antigens [201]. This subset of DCs in the lamina propria likely plays an important role in sensing intestinal pathogens and their products. However, the role of intestinal DCs in *C. difficile* toxin-induced intestinal inflammation has not yet been studied, although a recent study by Lee and colleagues has reported that TcdA promotes bone marrow-derived DC maturation and production of neutrophil-attracting chemokine [202]. 

Macrophages play an important role in the pathogenesis of *C. difficile*-induced colitis [23,203]. In the normal colon, intestinal macrophages lie in close proximity to the surface epithelial cells. In *C. difficile* colitis, tissue macrophages may well become exposed to toxins, especially following colonic micro-ulceration [29]. Macrophages are key sources of proinflammatory mediators including prostaglandins, leukotriene B4, nitric oxide (NO), IL-1β and TNF-α [204]. Ng *et al.* reported that TcdA and TcdB trigger IL-1β release by macrophages via activating an ASC (apoptosis-associated speck-like protein containing a CARD)-containing inflammsome, contributing to toxin-induced inflammation and damage *in vivo* [147]. Macrophages are also a major source of IL-8 [23]. In addition to IL-8, both IL-1β and TNF-α are involved in the neutrophil recruitment during CDI [138,139]. In a rat model, TcdB induces neutrophil migration via the release of leukotrienes, (notably B4) and TNF-α from resident macrophages [138]. Lamina propria macrophages are also activated by substance P (SP), releasing TNF-α during acute TcdA-induced intestinal inflammation. Administration of SP receptor antagonists diminishes the TNF-α production by lamina propria macrophages isolated from rat TcdA-exposed ileal loops [142]. Human monocytes are precursors of tissue macrophages. TcdA and TcdB are both able to induce IL-1, IL-6, TNF-α secretion by human monocytes [24]. In addition, TcdA can induce IL-8 secretion in peripheral blood monocytes and in the monocyte cell line THP-1 [23,132,205].

### 5.5. Role of neuronal cells

There is clear evidence that primary sensory neurons are also involved in *C. difficile* toxin-induced intestinal inflammatory response. Activation of the sensory nerves and release of sensory neuropeptides, including SP and calcitonin gene-related peptide (CGRP), are important in mediating and amplifying toxin-induced inflammatory signal [4,26]. Pothoulakis and colleagues demonstrated that SP and CGRP are elevated in the cell bodies of spinal cord dorsal root ganglia (DRG) and intestinal mucosa following TcdA-exposure of rat ileal loops [142,143]. Administration of capsaicin, a neurotoxin that desensitizes sensory nerve endings attenuated the toxin response. Moreover, SP receptor (*i.e.*, NK-1) and CGRP antagonists dramatically reduced TcdA-induced fluid secretion, mucosal permeability, and proinflammatory cytokine release [206]. In support of these findings, mice lacking NK-1 receptors were substantially desensitized to TcdA-induced enteropathy [207]. Using similar approaches this group also demonstrated that neuron peptide Corticotropin-releasing hormone (CRH) is required in TcdA mediated intestinal inflammation [208,209,210].

Melanin-concentrating hormone (MCH) is a hypothalamic orexigenic neuropeptide that regulates energy balance. Pothoulakis and colleagues found that TcdA induced upregulation of MCH and its receptor MCHR1 in the human intestinal xenograft model and of MCHR1 in colonocytes. Treatment of colonocytes with MCH upregulates the transcription of IL-8. Moreover, MCH-deficient mice develop an attenuated TcdA-mediated intestinal inflammation and secretion [148]. 

Neurotensin (NT) is another neuropeptide mediator in TcdA-induced enteritis [144]. NT is a peptide primarily synthesized in the brain and GI tract, and its expression is rapidly upregulated following toxin-induced enteropathy in the rat. The NT receptors are similarly upregulated by TcdA, and NT receptor antagonist inhibits TcdA-induced fluid secretion, mucosal permeability, and mast cell activation. Interestingly, NT-mediated degranulation of mucosal mast cells *in vitro* is inhibited by a SP receptor antagonist, suggesting a functional communication between NT and SP in TcdA-mediated enterotoxicity.

Neunlist *et al.* investigated the role of human submucosal neurons in TcdB-mediated responses. In their experiment setup, isolated segments of human colon were placed in an organ culture for 3 h in the presence of TcdB or IL-1β, and whole mounts of internal submucosal plexus were stained with antibodies against c-Fos, neuron-specific enolase (NSE), vasoactive intestinal polypeptide (VIP), and substance P (SP). The membrane potential (Vm) response of submucosal neurons to the local application of toxin B and IL-1β was determined by a multisite optical recording technique. Using this unique approach, they found that TcdB activates human VIP-positive submucosal neurons, partially via an IL-1β-dependent pathway [145]. Submucosal nervous system was also reported to be involved in IL-8 secretion in an IL-1β-dependent manner in TcdB-treated human intestinal tissues [135]. 

Further support for a neuronal involvement in CDI is provided by a study demonstrating the involvements of protease-activated receptor 2 (PAR2) in TcdA-induced enteropathy [146]. PAR2 belongs to a family of G protein-coupled receptors that are activated by proteolytic cleavage within their extracellular *N-*terminal domain. Within the intestine, PAR2 is expressed on enterocytes as well as on neurons, monocytes, endothelial cells, and neutrophils. Trypsins and tryptase are prominent agonists of PAR2 in the gastrointestinal tract. PAR2 activation is associated with pro-inflammatory effects, many of which show similarity to those induced by TcdA. In this study, PAR2 deletion or pretreatment of rat ileal loops with tryptase inhibitors decreases the TcdA-induced enteropathy. In addition, TcdA injection into ileal loops increases the expression of PAR2 and trypsin IV on epithelial cells. The authors also showed that tryptase and trypsin isozymes induce ileitis, which can be prevented by pretreatment of the ileal loops with NK-1 receptor antagonist. Therefore, PAR2 and its activators are proinflammatory in TcdA-induced enteritis and PAR2 causes inflammation through a neurogenic mechanism.

Another potential mediator of neuronal-mediated enteropathy in response to TcdA is leukotriene B4 (LTB_4_). Using rat ileal loops, McVey and Vigna showed that TcdA induces LTB_4_ secretion, a mediator that can cause a similar ileitis as TcdA [141]. They reported that the inflammatory effects of TcdA are blocked by inhibiting LTB_4_ synthesis, and that the inflammatory effect of LTB_4_ depends on the activation of the transient receptor potential vanilloid (TRPV1). TRPV1 is expressed on capsaicin-sensitive primary neuronal afferents of the DRG, and has been shown to partially mediate TcdA-induced inflammatory response in the rat intestine. In addition, this group found that LTB_4_ stimulates TRPV1-mediated endogenous SP release. Pretreatment of the ileum with NK-1 receptor antagonist blocks LTB_4_-induced SP action and inflammation. Therefore, LTB_4_ may mediate the inflammatory effects of TcdA via activation of TRPV1 receptor and by release of SP.

### 5.6. Role of other toxin-induced mediators in CDI

The acute intestinal inflammatory response to TcdA in animal models (mostly demonstrated in ileal loops of mice, rats, and rabbits), have demonstrated a clear role for TcdA in modulating inflammatory events. This inflammation is also implicated in the intestinal hyper-secretion and tissue damage. In addition to the mediators described above, several other classes of signaling molecules have been implicated in CDI. 

#### 5.6.1. Reactive oxygen species (ROS)

Qiu and colleagues reported that ROS participates in TcdA-induced enteritis in rats [149]. ROS and nitric oxide species have been implicated in the pathogenesis of experimental colitis in animal models and in idiopathic inflammatory bowel disease of humans. In this study, the authors showed that intraluminal TcdA causes a significant increase in hydroxyl radical and hydrogen peroxide production. The production of ROS is inhibited by pretreatment with either DMSO, a ROS scavenger, or with superoxide dismutase (SOD), an inhibitor of ROS. It appears that the mucosal xanthine oxidase is not directly involved in TcdA-associated intestinal response, suggesting ROS released during TcdA enteritis is primarily from neutrophils invading in the inflamed bowel segment. ROS involvement in TcdA-induced intestinal inflammation is also reported in a more recent study [150]. Moreover, nitric oxide species have been shown to play a protective role in TcdA-induced enteritis in rats [211]. Our group has recently demonstrated that a component of this nitric oxide based protection against TcdA is mediated by S-nitrosothiol intermediates generated during CDI (unpublished findings). The study by Hirota *et al.* has confirmed the importance of nitric oxide signals in protecting against TcdA-induced enteropathy, and has implicated NO regulation of HIF in this process. S-nitrosothiols (which are stable nitric oxide intermediates) are potent regulators of HIF-1 activity via S-nitrosylation of this transcription factor.

#### 5.6.2. Lipid inflammatory mediators

Lipid inflammatory mediators are also implicated in the initiation and perpetuation of TcdA-induced inflammation. Alcantara *et al.* demonstrated that inhibition of cyclooxygenase-2 (COX-2) blocks the TcdA-induced inflammatory response in rabbit ileal loops [153]. Kim *et al.* have shown that TcdA induces COX-2 expression and releases prostaglandin E2 (PGE2) in a dose- and time-dependent manner in cultured human colonocytes and in human intestinal xenografts. The main signaling pathway for TcdA-induced human COX-2 involves reactive oxygen species (ROS)-mediated activation of p38 MAPK, stress-activated protein kinase-1 (MSK-1), COX-2 cAMP-responsive element binding protein (CREB), and activating transcription factor 1 (ATF-1) [150]. The conclusion was that TcdA triggers ileal inflammation and fluid secretion via induction of COX-2 and release of PGE_2_. In line with this findings, Meyer *et al.* recently reported that TcdB directly stimulate human mast cells to synthesize PGE2/PGD2 (prostaglandin) in a p38 MAPK-dependent pathway [198]. Lima and colleagues demonstrated that blocking phospholipase A2 activity, which is involved in the initial biochemical synthesis pathway of inflammatory lipid mediators, prevents both the inflammatory response and enteropathy induced by TcdA in rabbit ileal loops and in human T-84 cells [212]. Platelet-activating factor (PAF) is a phospholipid synthesized and released by several tissues and cells such as neutrophils, eosinophils, basophils, mast cells and endothelial cells. PAF is one of the major metabolites of acylphospholipid after phospholipase A2 cleavage. In addition, PAF is a potent stimulus of phospholipase A2. Fonteles *et al.* demonstrated that PAF inhibitors are able to decrease the hyper-secretion and inflammation, indicating a regulatory role for PAF in TcdA-induced entropathy [155].

#### 5.6.3. Na+/H+ exchanger (NHE)

The Na^+^/H^+^ exchanger (NHE) family consists of eight identified members (NHE1-8), all of which are integral membrane proteins that catalyze electro-neutral exchange of alkali cations for H^+^. NHE3 is expressed in the apical membrane and in subapical endosomes of polarized epithelial cells. In intestine, NHE3 is thought to play a critical role in Na^+^ absorption and fluid homeostasis [213,214]. Recently, Hayashi *et al.* reported that TcdB causes the disappearance of NHE3 from apical surface in cultured epithelial cells, which, in addition to increased paracellular permeability, may also contribute to fluid secretion in CDI [156]. TcdB treatment leads to a pronounced inhibition of NHE3 activity, the translocation of NHE3 to a subapical endomembrane compartment, and dephosphorylation and extensive redistribution of Ezrin. The authors proposed that the inactivation of Rho GTPases by TcdB may cause the dissociation between NHE3 and the microvillar cytoskeleton by impairing the ability of ezrin to bridge the exchangers to actin, leading to NHE3 internalization and its consequent disappearance from the apical surface [156].

#### 5.6.4. Angiotensin II

The renin-angiotensin system (RAS) is a multi-organ system of proteins and receptors that is regulated by hormones and cytokines to control a number of mechanisms associated with fluid balance and vasoconstriction. Angiotensin II (Ang II) is the main active peptide in the renin-angiotensin system. It was known that Ang II was vasoactive, capable of stimulating vascular smooth muscle cells. AngII binding to the type 1 receptor (AT1) results in vasoconstriction, and its binding to the type 2 receptor (AT2) results in vasorelaxation. Both AT1 and AT2 are known to play a major role in the cardiovascular and renovascular systems that mediate inflammation, cell growth, and fibrosis. There has been considerable interest in the presence of a local angiotensin system in the gastrointestinal tract. Alcantara and colleagues investigated the role of Ang II in a rabbit ileal loop model of CDI [157]. They showed that Ang II receptor blockers not only inhibit TcdA-induced intestinal hyper-secretion, but also decrease the production of Ang II in the ileum. This raises the possibility that Ang II may participate in a positive feedback loop regulating the hyper-secretory response. 

#### 5.6.5. Epidermal growth factor receptor (EGFR)

Na *et al.* reported that TcdB signals an acute proinflammatory response in colonocytes via transactivation of the EGFR and activation of the Erk/MAP kinase pathway [158]. TcdB activates EGFR and Erk1/2, leading to their phosphorylation and subsequent IL-8 production by human colonocytes. Pretreatment of cells with an EGFR inhibitor or a neutralizing antibody blocks both TcdB-induced EGFR and Erk activation. Inhibition of EGFR and Erk activation significantly decreases TcdB-induced IL-8 expression. It is also possible that this EGFR activation results from inhibition of cellular Src-1 activity mediated by TcdA [169]. 

## 6. Concluding Remarks

**Figure 2:**
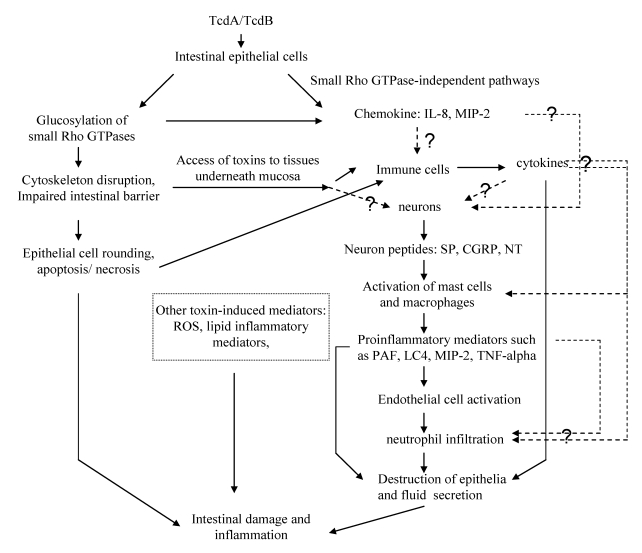
Mechanisms of TcdA- and TcdB-mediated enterotoxicity. TcdA and TcdB cause intestinal damage and inflammation by direct actions on intestinal epithelial cells, leading to chemokine release, cell rounding and apoptosis or necrosis. Alternatively, the toxins may target immune cells and neurons once the intestinal epithelial barrier is disrupted. The toxins may also act indirectly by stimulating cells to produce chemokine, proinflammatory cytokine, neuropeptides, and other mediators. IL-8: interleukin-8; MIP-2: macrophage inflammatory protein-2; SP: substance P; CGRP: calcitonin gene-related peptide; NT: neurotensin; PAF: Platelet-activating factor; LC4: Leukotriene C4; ROS: Reactive oxygen species.

Even though *C. difficile* infection has a broad spectrum of manifestations, it occurs almost exclusively in the large bowel and shows the characteristic microscopic and gross lesions, diarrhea and intestinal inflammation. Most of these distinguishing symptoms can be ascribed to inflammatory events mediated by TcdA and TcdB. Both TcdA and TcdB induce intestinal injury and inflammation through disruption of the intestinal epithelial barrier, induction of proinflammatory mediators and causing cell apoptosis or necrosis. Both TcdA and TcdB are glucosyltransferases that irreversibly inactivate small Rho GTPases, leading to disruption of cytoskeleton and tight junctions and subsequent cell rounding, detachment and cell death. In parallel, intestinal epithelial cells produce chemokines like IL-8 and adhesion molecules like ICAM-1, which further lead to the neutrophil adhesion, infiltration, and subsequent mucosal inflammation. The compromised mucosal barrier may allow toxins to directly act on immune cells and neurons to induce the secretion of proinflammatory cytokines like IL-1β, TNF-α, neuropeptides, and many other mediators. In addition, toxins by themselves or synergistically with other mediators can activate apoptotic or necrotic pathways in epithelial, immune cells, contributing to mucosal damage. All these events produce a complex pathophysiological response leading to intestinal inflammation (Figure 2). 

Our understanding of the enterotoxicity of TcdA and TcdB and their mechanisms of action has been expanded based on many exciting developments in this field. We can only highlight several pioneering work defining our views on *C. difficile* toxin enterotoxicity. It was recognized for a long time that TcdB was a cytotoxin, since purified TcdB alone fails to induce intestinal damage in animal models. Our group for the first time demonstrated the enterotoxicity of TcdB in a well designed human intestinal xenograft model [20]. Most recently Lyras and colleagues elegantly generated isogenic *C. difficile* strain in which *tcdA* or *tcdB* gene was inactivated. They demonstrated that TcdB plays a more important role than TcdA in causing CDI in hamsters [30]. In line with this TcdA^−^TcdB^+^ strains have been reported to cause human CDI [90]. While the determination of 3D structures of toxin fragments will is helpful to understand the bioactivities of the toxins, the scientists in the field are expecting to have high-resolution 3D structure of holotoxins. The identification of the autocatalytic cleavage of TcdB is another big stride in advancing our knowledge on the molecular mode of action of the toxins [115], which will help to rationalize new therapies targeting toxins’ autocatalytic activity.

Despite the progress that we have made in understanding the molecular mode of action of the two toxins, many fundamental questions remain unanswered. Are there specific receptors for TcdA and TcdB? And if so, what are they? Why do TcdA and TcdB differ considerably with respect to their biological activities while they share high sequences homology and similar domain structures, and act in a similar molecular mode? What are the respective roles of these two toxins in the host inflammatory response and pathogenesis? Given the increasing importance of CDI in public health, significant more research is necessary to fill these gaps in our knowledge in order to better tackle the disease. 

## References

[1] Cloud J., Kelly C.P. (2007). Update on *Clostridium difficile* associated disease. Curr. Opin. Gastroenterol..

[2] Elliott B., Chang B.J., Golledge C.L., Riley T.V. (2007). *Clostridium difficile*-associated diarrhoea. Intern. Med. J..

[3] Kelly C.P., Pothoulakis C., LaMont J.T. (1994). *Clostridium difficile* colitis. N. Engl. J. Med..

[4] Voth D.E., Ballard J.D. (2005). *Clostridium difficile* toxins: mechanism of action and role in disease. Clin. Microbiol. Rev..

[5] Carter G.P., Lyras D., Allen D.L., Mackin K.E., Howarth P.M., O'Connor J.R., Rood J.I. (2007). Binary toxin production in *Clostridium difficile* is regulated by CdtR, a LytTR family response regulator. J. Bacteriol..

[6] McMaster-Baxter N.L., Musher D.M. (2007). *Clostridium difficile*: recent epidemiologic findings and advances in therapy. Pharmacotherapy.

[7] Blossom D.B., McDonald L.C. (2007). The challenges posed by reemerging *Clostridium difficile* infection. Clin. Infect. Dis..

[8] Stare B.G., Delmee M., Rupnik M. (2007). Variant forms of the binary toxin CDT locus and tcdC gene in *Clostridium difficile* strains. J. Med. Microbiol..

[9] Geric B., Carman R.J., Rupnik M., Genheimer C.W., Sambol S.P., Lyerly D.M., Gerding D.N., Johnson S. (2006). Binary toxin-producing, large clostridial toxin-negative *Clostridium difficile* strains are enterotoxic but do not cause disease in hamsters. J. Infect. Dis..

[10] Schwan C., Stecher B., Tzivelekidis T., van Ham M., Rohde M., Hardt W.D., Wehland J., Aktories K. (2009). *Clostridium difficile* toxin CDT induces formation of microtubule-based protrusions and increases adherence of bacteria. PLoS Pathog..

[11] Borriello S.P., Davies H.A., Kamiya S., Reed P.J., Seddon S. (1990). Virulence factors of *Clostridium difficile*. Rev. Infect. Dis..

[12] Seddon S.V., Hemingway I., Borriello S.P. (1990). Hydrolytic enzyme production by *Clostridium difficile* and its relationship to toxin production and virulence in the hamster model. J. Med. Microbiol..

[13] Borriello S.P. (1998). Pathogenesis of *Clostridium difficile* infection. J. Antimicrob. Chemother..

[14] Calabi E., Calabi F., Phillips A.D., Fairweather N.F. (2002). Binding of *Clostridium difficile* surface layer proteins to gastrointestinal tissues. Infect. Immun..

[15] O'Brien J.B., McCabe M.S., Athie-Morales V., McDonald G.S., Ni Eidhin D.B., Kelleher D.P. (2005). Passive immunisation of hamsters against *Clostridium difficile* infection using antibodies to surface layer proteins. FEMS Microbiol. Lett..

[16] Halabi-Cabezon I., Huelsenbeck J., May M., Ladwein M., Rottner K., Just I., Genth H. (2008). Prevention of the cytopathic effect induced by *Clostridium difficile* Toxin B by active Rac1. FEBS Lett..

[17] Gerhard R., Nottrott S., Schoentaube J., Tatge H., Olling A., Just I. (2008). Glucosylation of Rho GTPases by *Clostridium difficile* toxin A triggers apoptosis in intestinal epithelial cells. J. Med. Microbiol..

[18] Brito G.A., Fujji J., Carneiro-Filho B.A., Lima A.A., Obrig T., Guerrant R.L. (2002). Mechanism of *Clostridium difficile* toxin A-induced apoptosis in T84 cells. J. Infect. Dis..

[19] Qa'Dan M., Ramsey M., Daniel J., Spyres L.M., Safiejko-Mroczka B., Ortiz-Leduc W., Ballard J.D. (2002). *Clostridium difficile* toxin B activates dual caspase-dependent and caspase-independent apoptosis in intoxicated cells. Cell. Microbiol..

[20] Savidge T.C., Pan W.H., Newman P., O'Brien M., Anton P.M., Pothoulakis C. (2003). *Clostridium difficile* toxin B is an inflammatory enterotoxin in human intestine. Gastroenterology.

[21] Kim J.M., Kim J.S., Jun H.C., Oh Y.K., Song I.S., Kim C.Y. (2002). Differential expression and polarized secretion of CXC and CC chemokines by human intestinal epithelial cancer cell lines in response to *Clostridium difficile* toxin A. Microbiol. Immunol..

[22] Ng E.K., Panesar N., Longo W.E., Shapiro M.J., Kaminski D.L., Tolman K.C., Mazuski J.E. (2003). Human intestinal epithelial and smooth muscle cells are potent producers of IL-6. Mediators Inflamm..

[23] Linevsky J.K., Pothoulakis C., Keates S., Warny M., Keates A.C., Lamont J.T., Kelly C.P. (1997). IL-8 release and neutrophil activation by *Clostridium difficile* toxin-exposed human monocytes. Am. J. Physiol..

[24] Flegel W.A., Muller F., Daubener W., Fischer H.G., Hadding U., Northoff H. (1991). Cytokine response by human monocytes to *Clostridium difficile* toxin A and toxin B. Infect. Immun..

[25] Kelly C.P., Becker S., Linevsky J.K., Joshi M.A., O'Keane J.C., Dickey B.F., LaMont J.T., Pothoulakis C. (1994). Neutrophil recruitment in *Clostridium difficile* toxin A enteritis in the rabbit. J. Clin. Invest..

[26] Pothoulakis C., Lamont J.T. (2001). Microbes and microbial toxins: paradigms for microbial-mucosal interactions II. The integrated response of the intestine to *Clostridium difficile* toxins. Am. J. Physiol. Gastrointest. Liver Physiol..

[27] Lyerly D.M., Lockwood D.E., Richardson S.H., Wilkins T.D. (1982). Biological activities of toxins A and B of *Clostridium difficile*. Infect. Immun..

[28] Lyerly D.M., Saum K.E., MacDonald D.K., Wilkins T.D. (1985). Effects of *Clostridium difficile* toxins given intragastrically to animals. Infect. Immun..

[29] Riegler M., Sedivy R., Pothoulakis C., Hamilton G., Zacherl J., Bischof G., Cosentini E., Feil W., Schiessel R., LaMont J.T. (1995). *Clostridium difficile* toxin B is more potent than toxin A in damaging human colonic epithelium *in vitro*. J. Clin. Invest..

[30] Lyras D., O'Connor J.R., Howarth P.M., Sambol S.P., Carter G.P., Phumoonna T., Poon R., Adams V., Vedantam G., Johnson S., Gerding D.N., Rood J.I. (2009). Toxin B is essential for virulence of *Clostridium difficile*. Nature.

[31] Shin B.M., Kuak E.Y., Yoo S.J., Shin W.C., Yoo H.M. (2008). Emerging toxin A-B+ variant strain of *Clostridium difficile* responsible for pseudomembranous colitis at a tertiary care hospital in Korea. Diagn. Microbiol. Infect. Dis..

[32] Hamm E.E., Voth D.E., Ballard J.D. (2006). Identification of *Clostridium difficile* toxin B cardiotoxicity using a zebrafish embryo model of intoxication. Proc. Natl. Acad. Sci. USA.

[33] Pavliakova D., Moncrief J.S., Lyerly D.M., Schiffman G., Bryla D.A., Robbins J.B., Schneerson R. (2000). *Clostridium difficile* recombinant toxin A repeating units as a carrier protein for conjugate vaccines: studies of pneumococcal type 14, *Escherichia coli* K1, and Shigella flexneri type 2a polysaccharides in mice. Infect. Immun..

[34] He X., Wang J., Steele J., Sun X., Nie W., Tzipori S., Feng H. (2009). An ultrasensitive rapid immunocytotoxicity assay for detecting *Clostridium difficile* toxins. J. Microbiol. Methods.

[35] Steele J., Feng H., Parry N., Tzipori S. (2010). Piglet models of acute or chronic *Clostridium difficile* illness. J. Infect. Dis..

[36] Roberts K., Smith C.F., Snelling A.M., Kerr K.G., Banfield K.R., Sleigh P.A., Beggs C.B. (2008). Aerial dissemination of *Clostridium difficile* spores. BMC Infect. Dis..

[37] Dubberke E.R., Reske K.A., Noble-Wang J., Thompson A., Killgore G., Mayfield J., Camins B., Woeltje K., McDonald J.R., McDonald L.C., Fraser V.J. (2007). Prevalence of *Clostridium difficile* environmental contamination and strain variability in multiple health care facilities. Am. J. Infect. Control.

[38] Bartlett J.G. (2006). Narrative review: the new epidemic of *Clostridium difficile*-associated enteric disease. Ann. Intern. Med..

[39] Johnson S., Kent S.A., O'Leary K.J., Merrigan M.M., Sambol S.P., Peterson L.R., Gerding D.N. (2001). Fatal pseudomembranous colitis associated with a variant *Clostridium difficile* strain not detected by toxin A immunoassay. Ann. Intern. Med..

[40] Jacob S.S., Sebastian J.C., Hiorns D., Jacob S., Mukerjee P.K. (2004). *Clostridium difficile* and acute respiratory distress syndrome. Heart Lung.

[41] Dobson G., Hickey C., Trinder J. (2003). *Clostridium difficile* colitis causing toxic megacolon, severe sepsis and multiple organ dysfunction syndrome. Intensive Care Med..

[42] Cunney R.J., Magee C., McNamara E., Smyth E.G., Walshe J. (1998). *Clostridium difficile* colitis associated with chronic renal failure. Nephrol. Dial. Transplant..

[43] Sakurai T., Hajiro K., Takakuwa H., Nishi A., Aihara M., Chiba T. (2001). Liver abscess caused by *Clostridium difficile*. Scand. J. Infect. Dis..

[44] Zar F., Bakkanagari S., Moorthi K.M.L.S.T., Davis M. (2007). A Comparison of Vancomycin and Metronidazole for the Treatment of *Clostridium difficile*—Associated Diarrhea, Stratified by Disease Severity. Clin. Infect. Dis..

[45] Barbut F., Richard A., Hamadi K., Chomette V., Burghoffer B., Petit J.C. (2000). Epidemiology of recurrences or reinfections of *Clostridium difficile*-associated diarrhea. J. Clin. Microbiol..

[46] Tonna I., Welsby P.D. (2005). Pathogenesis and treatment of *Clostridium difficile* infection. Postgrad. Med. J..

[47] McVay C.S., Rolfe R.D. (2000). *In vitro* and *in vivo* activities of nitazoxanide against *Clostridium difficile*. Antimicrob. Agents Chemother..

[48] Anton P.M., O'Brien M., Kokkotou E., Eisenstein B., Michaelis A., Rothstein D., Paraschos S., Kelly C.P., Pothoulakis C. (2004). Rifalazil treats and prevents relapse of *Clostridium difficile*-associated diarrhea in hamsters. Antimicrob. Agents Chemother..

[49] Hinkson P.L., Dinardo C., DeCiero D., Klinger J.D., Barker R.H. (2008). Tolevamer, an anionic polymer, neutralizes toxins produced by the BI/027 strains of *Clostridium difficile*. Antimicrob. Agents Chemother..

[50] Taylor C.P., Tummala S., Molrine D., Davidson L., Farrell R.J., Lembo A., Hibberd P.L., Lowy I., Kelly C.P. (2008). Open-label, dose escalation phase I study in healthy volunteers to evaluate the safety and pharmacokinetics of a human monoclonal antibody to *Clostridium difficile* toxin A. Vaccine.

[51] Kotloff K.L., Wasserman S.S., Losonsky G.A., Thomas W., Nichols R., Edelman R., Bridwell M., Monath T.P. (2001). Safety and immunogenicity of increasing doses of a *Clostridium difficile* toxoid vaccine administered to healthy adults. Infect. Immun..

[52] Sougioultzis S., Kyne L., Drudy D., Keates S., Maroo S., Pothoulakis C., Giannasca P.J., Lee C.K., Warny M., Monath T.P., Kelly C.P. (2005). *Clostridium difficile* toxoid vaccine in recurrent *C. difficile*-associated diarrhea. Gastroenterology.

[53] Archibald L.K., Banerjee S.N., Jarvis W.R. (2004). Secular trends in hospital-acquired *Clostridium difficile* disease in the United States, 1987–2001. J. Infect. Dis..

[54] Loo V.G., Poirier L., Miller M.A., Oughton M., Libman M.D., Michaud S., Bourgault A.M., Nguyen T., Frenette C., Kelly M., Vibien A., Brassard P., Fenn S., Dewar K., Hudson T.J., Horn R., Rene P., Monczak Y., Dascal A. (2005). A predominantly clonal multi-institutional outbreak of *Clostridium difficile*-associated diarrhea with high morbidity and mortality. N. Engl. J. Med..

[55] McDonald L.C., Killgore G.E., Thompson A., Owens R.C., Kazakova S.V., Sambol S.P., Johnson S., Gerding D.N. (2005). An epidemic, toxin gene-variant strain of *Clostridium difficile*. N. Engl. J. Med..

[56] Larson H.E., Barclay F.E., Honour P., Hill I.D. (1982). Epidemiology of *Clostridium difficile* in infants. J. Infect. Dis..

[57] Al-Jumaili I.J., Shibley M., Lishman A.H., Record C.O. (1984). Incidence and origin of *Clostridium difficile* in neonates. J. Clin. Microbiol..

[58] Boenning D.A., Fleisher G.R., Campos J.M., Hulkower C.W., Quinlan R.W. (1982). *Clostridium difficile* in a pediatric outpatient population. Pediatr. Infect. Dis..

[59] Bolton R.P., Tait S.K., Dear P.R., Losowsky M.S. (1984). Asymptomatic neonatal colonisation by *Clostridium difficile*. Arch. Dis. Child..

[60] Collignon A., Cotte-Laffitte J., Quero A.M., Torlotin J.C. (1986). *Clostridium difficile* and its cytotoxin in the stools of young hospitalized children. Influence of antibiotic treatment. Pathol. Biol. (Paris).

[61] Libby J.M., Donta S.T., Wilkins T.D. (1983). *Clostridium difficile* toxin A in infants. J. Infect. Dis..

[62] Chang T.W., Sullivan N.M., Wilkins T.D. (1986). Insusceptibility of fetal intestinal mucosa and fetal cells to *Clostridium difficile* toxins. Acta Pharmacol. Sin..

[63] Giesemann T., Guttenberg G., Aktories K. (2008). Human alpha-defensins inhibit *Clostridium difficile* toxin B. Gastroenterology.

[64] Abrams G.D., Allo M., Rifkin G.D., Fekety R., Silva J. (1980). Mucosal damage mediated by clostridial toxin in experimental clindamycin-associated colitis. Gut.

[65] Czuprynski C.J., Johnson W.J., Balish E., Wilkins T. (1983). Pseudomembranous colitis in *Clostridium difficile*-monoassociated rats. Infect. Immun..

[66] Fekety R., Silva J., Toshniwal R., Allo M., Armstrong J., Browne R., Ebright J., Rifkin G. (1979). Antibiotic-associated colitis: effects of antibiotics on *Clostridium difficile* and the disease in hamsters. Rev. Infect. Dis..

[67] Knoop F.C. (1979). Clindamycin-associated enterocolitis in guinea pigs: evidence for a bacterial toxin. Infect. Immun..

[68] Chang T.W., Bartlett J.G., Gorbach S.L., Onderdonk A.B. (1978). Clindamycin-induced enterocolitis in hamsters as a model of pseudomembranous colitis in patients. Infect. Immun..

[69] Corthier G., Dubos F., Raibaud P. (1985). Modulation of cytotoxin production by *Clostridium difficile* in the intestinal tracts of gnotobiotic mice inoculated with various human intestinal bacteria. Appl. Environ. Microbiol..

[70] Lusk R.H., Fekety R., Silva J., Browne R.A., Ringler D.H., Abrams G.D. (1978). Clindamycin-induced enterocolitis in hamsters. J. Infect. Dis..

[71] Price A.B., Larson H.E., Crow J. (1979). Morphology of experimental antibiotic-associated enterocolitis in the hamster: a model for human pseudomembranous colitis and antibiotic-associated diarrhoea. Gut.

[72] Rehg J.E., Pakes S.P. (1982). Implication of *Clostridium difficile* and *Clostridium perfringens* iota toxins in experimental lincomycin-associated colitis of rabbits. Lab. Anim. Sci..

[73] Sugiyama T., Mukai M., Yamashita R., Sunakawa K. (1985). Experimental models of *Clostridium difficile* enterocolitis in gnotobiotic mice. Prog. Clin. Biol. Res..

[74] Chen X., Katchar K., Goldsmith J.D., Nanthakumar N., Cheknis A., Gerding D.N., Kelly C.P. (2008). A mouse model of *Clostridium difficile*-associated disease. Gastroenterology.

[75] Keel M.K., Songer J.G. (2006). The comparative pathology of *Clostridium difficile*-associated disease. Vet. Pathol..

[76] Rolfe R.D., Iaconis J.P. (1983). Intestinal colonization of infant hamsters with *Clostridium difficile*. Infect. Immun..

[77] Lyerly D.M., Krivan H.C., Wilkins T.D. (1988). *Clostridium difficile*: its disease and toxins. Clin. Microbiol. Rev..

[78] Mitchell T.J., Ketley J.M., Haslam S.C., Stephen J., Burdon D.W., Candy D.C., Daniel R. (1986). Effect of toxin A and B of *Clostridium difficile* on rabbit ileum and colon. Gut.

[79] Lima A.A., Lyerly D.M., Wilkins T.D., Innes D.J., Guerrant R.L. (1988). Effects of *Clostridium difficile* toxins A and B in rabbit small and large intestine *in vivo* and on cultured cells *in vitro*. Infect. Immun..

[80] Bongaerts G.P., Lyerly D.M. (1994). Role of toxins A and B in the pathogenesis of *Clostridium difficile* disease. Infect. Immun..

[81] Ketley J.M., Mitchell T.J., Candy D.C., Burdon D.W., Stephen J. (1987). The effects of *Clostridium difficile* crude toxins and toxin A on ileal and colonic loops in immune and non-immune rabbits. J. Med. Microbiol..

[82] Kim P.H., Iaconis J.P., Rolfe R.D. (1987). Immunization of adult hamsters against *Clostridium difficile*-associated ileocecitis and transfer of protection to infant hamsters. Infect. Immun..

[83] Corthier G., Muller M.C., Wilkins T.D., Lyerly D., L'Haridon R. (1991). Protection against experimental pseudomembranous colitis in gnotobiotic mice by use of monoclonal antibodies against *Clostridium difficile* toxin A. Infect. Immun..

[84] Leav B.A., Blair B., Leney M., Knauber M., Reilly C., Lowy I., Gerding D.N., Kelly C.P., Katchar K., Baxter R., Ambrosino D., Molrine D. (2010). Serum anti-toxin B antibody correlates with protection from recurrent *Clostridium difficile* infection (CDI). Vaccine.

[85] Kyne L., Warny M., Qamar A., Kelly C.P. (2000). Asymptomatic carriage of *Clostridium difficile* and serum levels of IgG antibody against toxin A. N. Engl. J. Med..

[86] Bacon A.E., Fekety R. (1994). Immunoglobulin G directed against toxins A and B of *Clostridium difficile* in the general population and patients with antibiotic-associated diarrhea. Diagn. Microbiol. Infect. Dis..

[87] Libby J.M., Jortner B.S., Wilkins T.D. (1982). Effects of the two toxins of *Clostridium difficile* in antibiotic-associated cecitis in hamsters. Infect. Immun..

[88] Lonnroth I., Lange S. (1983). Toxin A of *Clostridium difficile*: production, purification and effect in mouse intestine. Acta Pathol. Microbiol. Immunol. Scand. B.

[89] von Eichel-Streiber C., Boquet P., Sauerborn M., Thelestam M. (1996). Large clostridial cytotoxins—a family of glycosyltransferases modifying small GTP-binding proteins. Trends Microbiol..

[90] Drudy D., Fanning S., Kyne L. (2007). Toxin A-negative, toxin B-positive *Clostridium difficile*. Int. J. Infect. Dis..

[91] Lawley T.D., Clare S., Walker A.W., Goulding D., Stabler R.A., Croucher N., Mastroeni P., Scott P., Raisen C., Mottram L., Fairweather N.F., Wren B.W., Parkhill J., Dougan G. (2009). Antibiotic treatment of *Clostridium difficile* carrier mice triggers a supershedder state, spore-mediated transmission, and severe disease in immunocompromised hosts. Infect. Immun..

[92] Lyerly D.M., Barroso L.A., Wilkins T.D., Depitre C., Corthier G. (1992). Characterization of a toxin A-negative, toxin B-positive strain of *Clostridium difficile*. Infect. Immun..

[93] Borriello S.P., Wren B.W., Hyde S., Seddon S.V., Sibbons P., Krishna M.M., Tabaqchali S., Manek S., Price A.B. (1992). Molecular, immunological, and biological characterization of a toxin A-negative, toxin B-positive strain of *Clostridium difficile*. Infect. Immun..

[94] Kink J.A., Williams J.A. (1998). Antibodies to recombinant *Clostridium difficile* toxins A and B are an effective treatment and prevent relapse of *C. difficile*-associated disease in a hamster model of infection. Infect. Immun..

[95] Babcock G.J., Broering T.J., Hernandez H.J., Mandell R.B., Donahue K., Boatright N., Stack A.M., Lowy I., Graziano R., Molrine D., Ambrosino D.M., Thomas W.D. (2006). Human monoclonal antibodies directed against toxins A and B prevent *Clostridium difficile*-induced mortality in hamsters. Infect. Immun..

[96] Eglow R., Pothoulakis C., Itzkowitz S., Israel E.J., O'Keane C.J., Gong D., Gao N., Xu Y.L., Walker W.A., LaMont J.T. (1992). Diminished *Clostridium difficile* toxin A sensitivity in newborn rabbit ileum is associated with decreased toxin A receptor. J. Clin. Invest..

[97] Rolfe R.D. (1991). Binding kinetics of *Clostridium difficile* toxins A and B to intestinal brush border membranes from infant and adult hamsters. Infect. Immun..

[98] Taylor N.S., Thorne G.M., Bartlett J.G. (1981). Comparison of two toxins produced by *Clostridium difficile*. Infect. Immun..

[99] Sutton P.A., Li S., Webb J., Solomon K., Brazier J., Mahida Y.R. (2008). Essential role of toxin A in *C. difficile* 027 and reference strain supernatant-mediated disruption of Caco-2 intestinal epithelial barrier function. Clin. Exp. Immunol..

[100] Just I., Gerhard R. (2004). Large clostridial cytotoxins. Rev. Physiol. Biochem. Pharmacol..

[101] Jank T., Aktories K. (2008). Structure and mode of action of clostridial glucosylating toxins: the ABCD model. Trends Microbiol..

[102] Ho J.G., Greco A., Rupnik M., Ng K.K. (2005). Crystal structure of receptor-binding *C*-terminal repeats from *Clostridium difficile* toxin A. Proc. Natl. Acad. Sci. USA.

[103] Greco A., Ho J.G., Lin S.J., Palcic M.M., Rupnik M., Ng K.K. (2006). Carbohydrate recognition by *Clostridium difficile* toxin A. Nat. Struct. Mol. Biol..

[104] Reinert D.J., Jank T., Aktories K., Schulz G.E. (2005). Structural basis for the function of *Clostridium difficile* toxin B. J. Mol. Biol..

[105] Pruitt R.N., Chagot B., Cover M., Chazin W.J., Spiller B., Lacy D.B. (2009). Structure-function analysis of inositol hexakisphosphate-induced autoprocessing in *Clostridium difficile* toxin A. J. Biol. Chem..

[106] Albesa-Jove D., Bertrand T., Carpenter E.P., Swain G.V., Lim J., Zhang J., Haire L.F., Vasisht N., Braun V., Lange A., von Eichel-Streiber C., Svergun D.I., Fairweather N.F., Brown K.A. (2010). Four distinct structural domains in *Clostridium difficile* toxin B visualized using SAXS. J. Mol. Biol..

[107] Krivan H.C., Clark G.F., Smith D.F., Wilkins T.D. (1986). Cell surface binding site for *Clostridium difficile* enterotoxin: evidence for a glycoconjugate containing the sequence Gal alpha 1-3Gal beta 1-4GlcNAc. Infect. Immun..

[108] Tucker K.D., Wilkins T.D. (1991). Toxin A of *Clostridium difficile* binds to the human carbohydrate antigens I, X, and Y. Infect. Immun..

[109] Rolfe R.D., Song W. (1993). Purification of a functional receptor for *Clostridium difficile* toxin A from intestinal brush border membranes of infant hamsters. Clin. Infect. Dis..

[110] Teneberg S., Lonnroth I., Torres Lopez J.F., Galili U., Halvarsson M.O., Angstrom J., Karlsson K.A. (1996). Molecular mimicry in the recognition of glycosphingolipids by Gal alpha 3 Gal beta 4 GlcNAc beta-binding *Clostridium difficile* toxin A, human natural anti alpha-galactosyl IgG and the monoclonal antibody Gal-13: characterization of a binding-active human glycosphingolipid, non-identical with the animal receptor. Glycobiology.

[111] Koike T., Kuzuya M., Asai T., Kanda S., Cheng X.W., Watanabe K., Banno Y., Nozawa Y., Iguchi A. (2000). Activation of MMP-2 by *Clostridium difficile* toxin B in bovine smooth muscle cells. Biochem. Biophys. Res. Commun..

[112] Jank T., Giesemann T., Aktories K. (2007). Rho-glucosylating *Clostridium difficile* toxins A and B: new insights into structure and function. Glycobiology.

[113] Na X., Kim H., Moyer M.P., Pothoulakis C., LaMont J.T. (2008). gp96 is a human colonocyte plasma membrane binding protein for *Clostridium difficile* toxin A. Infect. Immun..

[114] Stubbe H., Berdoz J., Kraehenbuhl J.P., Corthesy B. (2000). Polymeric IgA is superior to monomeric IgA and IgG carrying the same variable domain in preventing *Clostridium difficile* toxin A damaging of T84 monolayers. J. Immunol..

[115] Reineke J., Tenzer S., Rupnik M., Koschinski A., Hasselmayer O., Schrattenholz A., Schild H., von Eichel-Streiber C. (2007). Autocatalytic cleavage of *Clostridium difficile* toxin B. Nature.

[116] Karlsson K.A. (1995). Microbial recognition of target-cell glycoconjugates. Curr. Opin. Struct. Biol..

[117] Florin I., Thelestam M. (1983). Internalization of *Clostridium difficile* cytotoxin into cultured human lung fibroblasts. Biochim. Biophys. Acta.

[118] Florin I., Thelestam M. (1986). Lysosomal involvement in cellular intoxication with *Clostridium difficile* toxin B. Microb. Pathog..

[119] Henriques B., Florin I., Thelestam M. (1987). Cellular internalisation of *Clostridium difficile* toxin A. Microb. Pathog..

[120] Giesemann T., Jank T., Gerhard R., Maier E., Just I., Benz R., Aktories K. (2006). Cholesterol-dependent pore formation of *Clostridium difficile* toxin A. J. Biol. Chem..

[121] Qa'Dan M., Spyres L.M., Ballard J.D. (2000). pH-induced conformational changes in *Clostridium difficile* toxin B. Infect. Immun..

[122] Jank T., Giesemann T., Aktories K. (2007). *Clostridium difficile* glucosyltransferase toxin B-essential amino acids for substrate binding. J. Biol. Chem..

[123] Just I., Selzer J., Wilm M., von Eichel-Streiber C., Mann M., Aktories K. (1995). Glucosylation of Rho proteins by *Clostridium difficile* toxin B. Nature.

[124] Sehr P., Joseph G., Genth H., Just I., Pick E., Aktories K. (1998). Glucosylation and ADP ribosylation of rho proteins: effects on nucleotide binding, GTPase activity, and effector coupling. Biochemistry.

[125] Chaves-Olarte E., Freer E., Parra A., Guzman-Verri C., Moreno E., Thelestam M. (2003). R-Ras glucosylation and transient RhoA activation determine the cytopathic effect produced by toxin B variants from toxin A-negative strains of *Clostridium difficile*. J. Biol. Chem..

[126] Soehn F., Wagenknecht-Wiesner A., Leukel P., Kohl M., Weidmann M., von Eichel-Streiber C., Braun V. (1998). Genetic rearrangements in the pathogenicity locus of *Clostridium difficile* strain 8864—implications for transcription, expression and enzymatic activity of toxins A and B. Mol. Gen. Genet..

[127] Mehlig M., Moos M., Braun V., Kalt B., Mahony D.E., von Eichel-Streiber C. (2001). Variant toxin B and a functional toxin A produced by *Clostridium difficile* C34. FEMS Microbiol. Lett..

[128] Rupnik M. (2008). Heterogeneity of large clostridial toxins: importance of *Clostridium difficile* toxinotypes. FEMS Microbiol. Rev..

[129] Goncalves C., Decre D., Barbut F., Burghoffer B., Petit J.C. (2004). Prevalence and characterization of a binary toxin (actin-specific ADP-ribosyltransferase) from *Clostridium difficile*. J. Clin. Microbiol..

[130] Geric B., Johnson S., Gerding D.N., Grabnar M., Rupnik M. (2003). Frequency of binary toxin genes among *Clostridium difficile* strains that do not produce large clostridial toxins. J. Clin. Microbiol..

[131] Sundriyal A., Roberts A.K., Shone C.C., Acharya K.R. (2009). Structural basis for substrate recognition in the enzymatic component of ADP-ribosyltransferase toxin CDTa from *Clostridium difficile*. J. Biol. Chem..

[132] Mahida Y.R., Makh S., Hyde S., Gray T., Borriello S.P. (1996). Effect of *Clostridium difficile* toxin A on human intestinal epithelial cells: induction of interleukin 8 production and apoptosis after cell detachment. Gut.

[133] Castagliuolo I., Keates A.C., Wang C.C., Pasha A., Valenick L., Kelly C.P., Nikulasson S.T., LaMont J.T., Pothoulakis C. (1998). *Clostridium difficile* toxin A stimulates macrophage-inflammatory protein-2 production in rat intestinal epithelial cells. J. Immunol..

[134] Ishida Y., Maegawa T., Kondo T., Kimura A., Iwakura Y., Nakamura S., Mukaida N. (2004). Essential involvement of IFN-gamma in *Clostridium difficile* toxin A-induced enteritis. J. Immunol..

[135] Tixier E., Lalanne F., Just I., Galmiche J.P., Neunlist M. (2005). Human mucosa/submucosa interactions during intestinal inflammation: involvement of the enteric nervous system in interleukin-8 secretion. Cell. Microbiol..

[136] Kelly C.P., Keates S., Siegenberg D., Linevsky J.K., Pothoulakis C., Brady H.R. (1994). IL-8 secretion and neutrophil activation by HT-29 colonic epithelial cells. Am. J. Physiol..

[137] Canny G., Drudy D., Macmathuna P., O'Farrelly C., Baird A.W. (2006). Toxigenic *C. difficile* induced inflammatory marker expression by human intestinal epithelial cells is asymmetrical. Life Sci..

[138] Souza M.H., Melo-Filho A.A., Rocha M.F., Lyerly D.M., Cunha F.Q., Lima A.A., Ribeiro R.A. (1997). The involvement of macrophage-derived tumour necrosis factor and lipoxygenase products on the neutrophil recruitment induced by *Clostridium difficile* toxin B. Immunology.

[139] Rocha M.F., Maia M.E., Bezerra L.R., Lyerly D.M., Guerrant R.L., Ribeiro R.A., Lima A.A. (1997). *Clostridium difficile* toxin A induces the release of neutrophil chemotactic factors from rat peritoneal macrophages: role of interleukin-1beta, tumor necrosis factor alpha, and leukotrienes. Infect. Immun..

[140] Pothoulakis C., Castagliuolo I., LaMont J.T. (1998). Nerves and Intestinal Mast Cells Modulate Responses to Enterotoxins. News Physiol. Sci..

[141] McVey D.C., Vigna S.R. (2005). The role of leukotriene B4 in *Clostridium difficile* toxin A-induced ileitis in rats. Gastroenterology.

[142] Castagliuolo I., Keates A.C., Qiu B., Kelly C.P., Nikulasson S., Leeman S.E., Pothoulakis C. (1997). Increased substance P responses in dorsal root ganglia and intestinal macrophages during *Clostridium difficile* toxin A enteritis in rats. Proc. Natl. Acad. Sci. USA.

[143] Keates A.C., Castagliuolo I., Qiu B., Nikulasson S., Sengupta A., Pothoulakis C. (1998). CGRP upregulation in dorsal root ganglia and ileal mucosa during *Clostridium difficile* toxin A-induced enteritis. J. Biol. Chem..

[144] Castagliuolo I., Wang C.C., Valenick L., Pasha A., Nikulasson S., Carraway R.E., Pothoulakis C. (1999). Neurotensin is a proinflammatory neuropeptide in colonic inflammation. J. Clin. Invest..

[145] Neunlist M., Barouk J., Michel K., Just I., Oreshkova T., Schemann M., Galmiche J.P. (2003). Toxin B of *Clostridium difficile* activates human VIP submucosal neurons, in part via an IL-1beta-dependent pathway. Am. J. Physiol. Gastrointest. Liver Physiol..

[146] Cottrell G.S., Amadesi S., Pikios S., Camerer E., Willardsen J.A., Murphy B.R., Caughey G.H., Wolters P.J., Coughlin S.R., Peterson A., Knecht W., Pothoulakis C., Bunnett N.W., Grady E.F. (2007). Protease-activated receptor 2, dipeptidyl peptidase I, and proteases mediate *Clostridium difficile* toxin A enteritis. Gastroenterology.

[147] Ng J., Hirota S.A., Gross O., Li Y., Ulke-Lemee A., Potentier M.S., Schenck L.P., Vilaysane A., Seamone M.E., Feng H., Armstrong G.D., Tschopp J., Macdonald J.A., Muruve D.A., Beck P.L. (2010). *Clostridium difficile* toxin-induced inflammation and intestinal injury are mediated by the inflammasome. Gastroenterology.

[148] Kokkotou E., Espinoza D.O., Torres D., Karagiannides I., Kosteletos S., Savidge T., O'Brien M., Pothoulakis C. (2009). Melanin-concentrating hormone (MCH) modulates C difficile toxin A-mediated enteritis in mice. Gut.

[149] Qiu B., Pothoulakis C., Castagliuolo I., Nikulasson S., LaMont J.T. (1999). Participation of reactive oxygen metabolites in *Clostridium difficile* toxin A-induced enteritis in rats. Am. J. Physiol..

[150] Kim H., Rhee S.H., Kokkotou E., Na X., Savidge T., Moyer M.P., Pothoulakis C., LaMont J.T. (2005). *Clostridium difficile* toxin A regulates inducible cyclooxygenase-2 and prostaglandin E2 synthesis in colonocytes via reactive oxygen species and activation of p38 MAPK. J. Biol. Chem..

[151] He D., Sougioultzis S., Hagen S., Liu J., Keates S., Keates A.C., Pothoulakis C., Lamont J.T. (2002). *Clostridium difficile* toxin A triggers human colonocyte IL-8 release via mitochondrial oxygen radical generation. Gastroenterology.

[152] Roebuck K.A. (1999). Oxidant stress regulation of IL-8 and ICAM-1 gene expression: differential activation and binding of the transcription factors AP-1 and NF-kappaB (Review). Int. J. Mol. Med..

[153] Alcantara C., Stenson W.F., Steiner T.S., Guerrant R.L. (2001). Role of inducible cyclooxygenase and prostaglandins in *Clostridium difficile* toxin A-induced secretion and inflammation in an animal model. J. Infect. Dis..

[154] Lima A.A., Nascimento N.R., Fang G.D., Yotseff P., Toyama M.H., Guerrant R.L., Fonteles M.C. (2008). Role of phospholipase A(2) and tyrosine kinase in *Clostridium difficile* toxin A-induced disruption of epithelial integrity, histologic inflammatory damage and intestinal secretion. J. Appl. Toxicol..

[155] Fonteles M., Fang G., Thielman N.M., Yotseff P.S., Guerrant R.L. (1995). Role of platelet activating factor in the inflammatory and secretory effects of *Clostridium difficile* toxin A. J. Lipid Mediat. Cell Signal..

[156] Hayashi H., Szaszi K., Coady-Osberg N., Furuya W., Bretscher A.P., Orlowski J., Grinstein S. (2004). Inhibition and redistribution of NHE3, the apical Na+/H+ exchanger, by *Clostridium difficile* toxin B. J. Gen. Physiol..

[157] Alcantara C.S., Jin X.H., Brito G.A., Carneiro-Filho B.A., Barrett L.J., Carey R.M., Guerrant R.L. (2005). Angiotensin II subtype 1 receptor blockade inhibits *Clostridium difficile* toxin A-induced intestinal secretion in a rabbit model. J. Infect. Dis..

[158] Na X., Zhao D., Koon H.W., Kim H., Husmark J., Moyer M.P., Pothoulakis C., LaMont J.T. (2005). *Clostridium difficile* toxin B activates the EGF receptor and the ERK/MAP kinase pathway in human colonocytes. Gastroenterology.

[159] Feltis B.A., Wiesner S.M., Kim A.S., Erlandsen S.L., Lyerly D.L., Wilkins T.D., Wells C.L. (2000). *Clostridium difficile* toxins A and B can alter epithelial permeability and promote bacterial paracellular migration through HT-29 enterocytes. Shock.

[160] Aktories K., Just I. (1995). Monoglucosylation of low-molecular-mass GTP-binding Rho proteins by clostridial cytotoxins. Trends Cell Biol..

[161] Hecht G., Pothoulakis C., LaMont J.T., Madara J.L. (1988). *Clostridium difficile* toxin A perturbs cytoskeletal structure and tight junction permeability of cultured human intestinal epithelial monolayers. J. Clin. Invest..

[162] Hecht G., Koutsouris A., Pothoulakis C., LaMont J.T., Madara J.L. (1992). *Clostridium difficile* toxin B disrupts the barrier function of T84 monolayers. Gastroenterology.

[163] Johal S.S., Solomon K., Dodson S., Borriello S.P., Mahida Y.R. (2004). Differential effects of varying concentrations of *Clostridium difficile* toxin A on epithelial barrier function and expression of cytokines. J. Infect. Dis..

[164] Moore R., Pothoulakis C., LaMont J.T., Carlson S., Madara J.L. (1990). *C. difficile* toxin A increases intestinal permeability and induces Cl- secretion. Am. J. Physiol..

[165] Triadafilopoulos G., Pothoulakis C., O'Brien M.J., LaMont J.T. (1987). Differential effects of *Clostridium difficile* toxins A and B on rabbit ileum. Gastroenterology.

[166] Triadafilopoulos G., Pothoulakis C., Weiss R., Giampaolo C., Lamont J.T. (1989). Comparative study of *Clostridium difficile* toxin A and cholera toxin in rabbit ileum. Gastroenterology.

[167] Chen M.L., Pothoulakis C., LaMont J.T. (2002). Protein kinase C signaling regulates ZO-1 translocation and increased paracellular flux of T84 colonocytes exposed to *Clostridium difficile* toxin A. J. Biol. Chem..

[168] Teichert M., Tatge H., Schoentaube J., Just I., Gerhard R. (2006). Application of mutated *Clostridium difficile* toxin A for determination of glucosyltransferase-dependent effects. Infect. Immun..

[169] Kim H., Rhee S.H., Pothoulakis C., LaMont J.T. (2009). *Clostridium difficile* toxin A binds colonocyte Src causing dephosphorylation of focal adhesion kinase and paxillin. Exp. Cell Res..

[170] Fiorentini C., Fabbri A., Falzano L., Fattorossi A., Matarrese P., Rivabene R., Donelli G. (1998). *Clostridium difficile* toxin B induces apoptosis in intestinal cultured cells. Infect. Immun..

[171] Solomon K., Webb J., Ali N., Robins R.A., Mahida Y.R. (2005). Monocytes are highly sensitive to *Clostridium difficile* toxin A-induced apoptotic and nonapoptotic cell death. Infect. Immun..

[172] Mahida Y.R., Galvin A., Makh S., Hyde S., Sanfilippo L., Borriello S.P., Sewell H.F. (1998). Effect of *Clostridium difficile* toxin A on human colonic lamina propria cells: early loss of macrophages followed by T-cell apoptosis. Infect. Immun..

[173] Hippenstiel S., Schmeck B., N'Guessan P.D., Seybold J., Krull M., Preissner K., Eichel-Streiber C.V., Suttorp N. (2002). Rho protein inactivation induced apoptosis of cultured human endothelial cells. Am. J. Physiol. Lung Cell. Mol. Physiol..

[174] Huelsenbeck J., Dreger S., Gerhard R., Barth H., Just I., Genth H. (2007). Difference in the cytotoxic effects of toxin B from *Clostridium difficile* strain VPI 10463 and toxin B from variant *Clostridium difficile* strain 1470. Infect. Immun..

[175] Matarrese P., Falzano L., Fabbri A., Gambardella L., Frank C., Geny B., Popoff M.R., Malorni W., Fiorentini C. (2007). *Clostridium difficile* toxin B causes apoptosis in epithelial cells by thrilling mitochondria. Involvement of ATP-sensitive mitochondrial potassium channels. J. Biol. Chem..

[176] Pfeifer G., Schirmer J., Leemhuis J., Busch C., Meyer D.K., Aktories K., Barth H. (2003). Cellular uptake of *Clostridium difficile* toxin B. Translocation of the *N*-terminal catalytic domain into the cytosol of eukaryotic cells. J. Biol. Chem..

[177] Warny M., Keates A.C., Keates S., Castagliuolo I., Zacks J.K., Aboudola S., Qamar A., Pothoulakis C., LaMont J.T., Kelly C.P. (2000). p38 MAP kinase activation by *Clostridium difficile* toxin A mediates monocyte necrosis, IL-8 production, and enteritis. J. Clin. Invest..

[178] Warny M., Kelly C.P. (1999). Monocytic cell necrosis is mediated by potassium depletion and caspase-like proteases. Am. J. Physiol..

[179] Kim H., Kokkotou E., Na X., Rhee S.H., Moyer M.P., Pothoulakis C., Lamont J.T. (2005). *Clostridium difficile* toxin A-induced colonocyte apoptosis involves p53-dependent p21(WAF1/CIP1) induction via p38 mitogen-activated protein kinase. Gastroenterology.

[180] Chae S., Eckmann L., Miyamoto Y., Pothoulakis C., Karin M., Kagnoff M.F. (2006). Epithelial cell I kappa B-kinase beta has an important protective role in *Clostridium difficile* toxin A-induced mucosal injury. J. Immunol..

[181] Hirota S.A., Fines K., Ng J., Traboulsi D., Lee J., Ihara E., Li Y., Willmore W.G., Chung D., Scully M.M., Louie T., Medlicott S., Lejeune M., Chadee K., Armstrong G., Colgan S.P., Muruve D.A., Macdonald J.A., Beck P.L. (2010). Hypoxia-inducible factor signaling provides protection in *Clostridium difficile*-induced intestinal Injury. Gastroenterology.

[182] Johnson S., Gerding D.N. (1998). *Clostridium difficile*-associated diarrhea. Clin. Infect. Dis..

[183] Kelly C.P., LaMont J.T. (1998). *Clostridium difficile* infection. Annu. Rev. Med..

[184] Burakoff R., Zhao L., Celifarco A.J., Rose K.L., Donovan V., Pothoulakis C., Percy W.H. (1995). Effects of purified *Clostridium difficile* toxin A on rabbit distal colon. Gastroenterology.

[185] Springer T.A. (1994). Traffic signals for lymphocyte recirculation and leukocyte emigration: the multistep paradigm. Cell.

[186] Miller M.D., Krangel M.S. (1992). Biology and biochemistry of the chemokines: a family of chemotactic and inflammatory cytokines. Crit. Rev. Immunol..

[187] Hoch R.C., Schraufstatter I.U., Cochrane C.G. (1996). *In vivo*, *in vitro*, and molecular aspects of interleukin-8 and the interleukin-8 receptors. J. Lab. Clin. Med..

[188] Matsukawa A., Yoshimura T., Maeda T., Ohkawara S., Takagi K., Yoshinaga M.  (1995). Neutrophil accumulation and activation by homologous IL-8 in rabbits. IL-8 induces destruction of cartilage and production of IL-1 and IL-1 receptor antagonist *in vivo*. J. Immunol..

[189] Sun X., He X., Tzipori S., Gerhard R., Feng H. (2009). Essential role of the glucosyltransferase activity in *Clostridium difficile* toxin-induced secretion of TNF-alpha by macrophages. Microb. Pathog..

[190] Yeh C.Y., Lin C.N., Chang C.F., Lin C.H., Lien H.T., Chen J.Y., Chia J.S. (2008). *C*-terminal repeats of *Clostridium difficile* toxin A induce production of chemokine and adhesion molecules in endothelial cells and promote migration of leukocytes. Infect. Immun..

[191] Kim J.M., Lee J.Y., Yoon Y.M., Oh Y.K., Youn J., Kim Y.J. (2006). NF-kappa B activation pathway is essential for the chemokine expression in intestinal epithelial cells stimulated with *Clostridium difficile* toxin A. Scand. J. Immunol..

[192] Jefferson K.K., Smith M.F., Bobak D.A. (1999). Roles of intracellular calcium and NF-kappa B in the *Clostridium difficile* toxin A-induced up-regulation and secretion of IL-8 from human monocytes. J. Immunol..

[193] Wershil B.K., Castagliuolo I., Pothoulakis C. (1998). Direct evidence of mast cell involvement in *Clostridium difficile* toxin A-induced enteritis in mice. Gastroenterology.

[194] Torimoto K., Sato N., Okubo M., Yagihashi A., Wada Y., Hara I., Hayasaka H., Kikuchi K. (1990). Development of multiple necrotizing enteritis induced by a tumor necrosis factor-like cytokine from lipopolysaccharide-stimulated peritoneal macrophages in rats. Am. J. Pathol..

[195] Pothoulakis C., Sullivan R., Melnick D.A., Triadafilopoulos G., Gadenne A.S., Meshulam T., LaMont J.T. (1988). *Clostridium difficile* toxin A stimulates intracellular calcium release and chemotactic response in human granulocytes. Am. J. Pathol..

[196] Prepens U., Just I., von Eichel-Streiber C., Aktories K. (1996). Inhibition of Fc epsilon-RI-mediated activation of rat basophilic leukemia cells by *Clostridium difficile* toxin B (monoglucosyltransferase). J. Biol. Chem..

[197] Wex C.B., Koch G., Aktories K. (1997). Effects of *Clostridium difficile* toxin B on activation of rat peritoneal mast cells. Naunyn Schmiedebergs Arch. Pharmacol..

[198] Meyer G.K., Neetz A., Brandes G., Tsikas D., Butterfield J.H., Just I., Gerhard R. (2007). *Clostridium difficile* toxins A and B directly stimulate human mast cells. Infect. Immun..

[199] Steinman R.M. (1991). The dendritic cell system and its role in immunogenicity. Annu. Rev. Immunol..

[200] Sozzani S., Allavena P., Vecchi A., Mantovani A. (2000). Chemokines and dendritic cell traffic. J. Clin. Immunol..

[201] Niess J.H., Brand S., Gu X., Landsman L., Jung S., McCormick B.A., Vyas J.M., Boes M., Ploegh H.L., Fox J.G., Littman D.R., Reinecker H.C. (2005). CX3CR1-mediated dendritic cell access to the intestinal lumen and bacterial clearance. Science.

[202] Lee J.Y., Kim H., Cha M.Y., Park H.G., Kim Y.J., Kim I.Y., Kim J.M. (2009). *Clostridium difficile* toxin A promotes dendritic cell maturation and chemokine CXCL2 expression through p38, IKK, and the NF-kappaB signaling pathway. J. Mol. Med..

[203] Pothoulakis C., LaMont J.T. (1993). *Clostridium difficile* colitis and diarrhea. Gastroenterol. Clin. North Am..

[204] Melo Filho A.A., Souza M.H., Lyerly D.M., Cunha F.Q., Lima A.A., Ribeiro R.A. (1997). Role of tumor necrosis factor and nitric oxide in the cytotoxic effects of *Clostridium difficile* toxin A and toxin B on macrophages. Toxicon.

[205] Branka J.E., Vallette G., Jarry A., Bou-Hanna C., Lemarre P., Van P.N., Laboisse C.L. (1997). Early functional effects of *Clostridium difficile* toxin A on human colonocytes. Gastroenterology.

[206] Castagliuolo I., LaMont J.T., Letourneau R., Kelly C., O'Keane J.C., Jaffer A., Theoharides T.C., Pothoulakis C. (1994). Neuronal involvement in the intestinal effects of *Clostridium difficile* toxin A and Vibrio cholerae enterotoxin in rat ileum. Gastroenterology.

[207] Castagliuolo I., Riegler M., Pasha A., Nikulasson S., Lu B., Gerard C., Gerard N.P., Pothoulakis C. (1998). Neurokinin-1 (NK-1) receptor is required in *Clostridium difficile*- induced enteritis. J. Clin. Invest..

[208] Anton P.M., Gay J., Mykoniatis A., Pan A., O'Brien M., Brown D., Karalis K., Pothoulakis C. (2004). Corticotropin-releasing hormone (CRH) requirement in *Clostridium difficile* toxin A-mediated intestinal inflammation. Proc. Natl. Acad. Sci. USA.

[209] Kokkotou E., Torres D., Moss A.C., O'Brien M., Grigoriadis D.E., Karalis K., Pothoulakis C. (2006). Corticotropin-releasing hormone receptor 2-deficient mice have reduced intestinal inflammatory responses. J. Immunol..

[210] Wlk M., Wang C.C., Venihaki M., Liu J., Zhao D., Anton P.M., Mykoniatis A., Pan A., Zacks J., Karalis K., Pothoulakis C. (2002). Corticotropin-releasing hormone antagonists possess anti-inflammatory effects in the mouse ileum. Gastroenterology.

[211] Qiu B., Pothoulakis C., Castagliuolo I., Nikulasson Z., LaMont J.T. (1996). Nitric oxide inhibits rat intestinal secretion by *Clostridium difficile* toxin A but not Vibrio cholerae enterotoxin. Gastroenterology.

[212] Lima A.A., Nascimento N.R., Fang G.D., Yotseff P., Toyama M.H., Guerrant R.L., Fonteles M.C. (2008). Role of phospholipase A2 and tyrosine kinase in *Clostridium difficile* toxin A-induced disruption of epithelial integrity, histologic inflammatory damage and intestinal secretion. J. Appl. Toxicol..

[213] Orlowski J., Grinstein S. (1997). Na+/H+ exchangers of mammalian cells. J. Biol. Chem..

[214] Biemesderfer D., Pizzonia J., Abu-Alfa A., Exner M., Reilly R., Igarashi P., Aronson P.S. (1993). NHE3: a Na+/H+ exchanger isoform of renal brush border. J. Biol. Chem..

